# Long-range metal–metal cooperative nitrile activation in 1,8-diazaanthracene-supported dicopper complexes

**DOI:** 10.1039/d6cc03416k

**Published:** 2026-06-24

**Authors:** Arie J. H. Multem, Vishal Chugh, Annemijn M. van Koten, Mick J. Zomer, Martin Lutz, Daniël L. J. Broere

**Affiliations:** a Organic Chemistry and Catalysis, Institute for Sustainable and Circular Chemistry, Faculty of Science, Utrecht University, Universiteitsweg 99 3584 CG Utrecht The Netherlands d.l.j.broere@uu.nl; b Structural Biochemistry Bijvoet Centre for Biomolecular Research, Faculty of Science, Utrecht University, Universiteitsweg 99 3584 CG Utrecht The Netherlands

## Abstract

A 1,8-diazaanthracene based ligand (^*t*Bu^PN-NP) is introduced as a platform that can combine metal–ligand cooperativity with long range metal–metal cooperativity.

## Graphical abstract

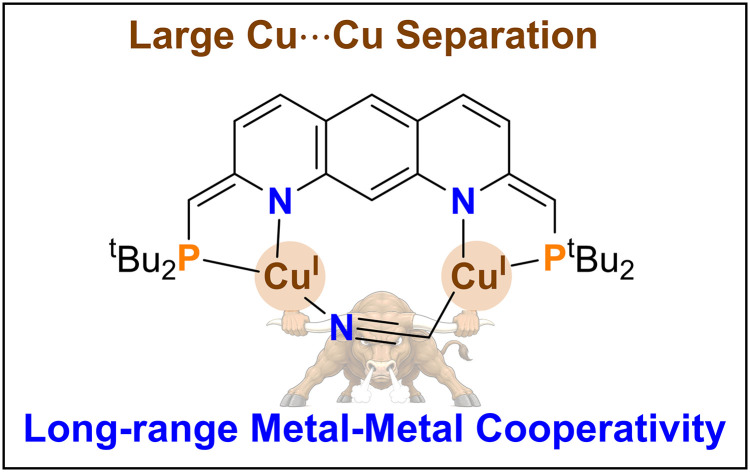

Homogeneous transition metal catalysts play a central role in modern synthetic chemistry, enabling the selective formation of C–C, C–N, and C–O bonds.^1,2^ Historically, most advances in homogeneous catalysis have relied on mononuclear complexes, where careful ligand design allows tuning of the steric and electronic environment around the reactive metal center to optimize catalytic performance.^3^ Over the past decades, increasing attention has shifted toward cooperative approaches, in which multiple components of a metal complex participate directly in substrate activation and bond-forming processes. Among the various forms of cooperative approaches, metal–ligand cooperativity (MLC) or metal–metal cooperativity (MMC) have emerged as particularly powerful strategies.^4^ For example, ligands capable of undergoing reversible aromatization/dearomatization can participate directly in bond activation through MLC, enabling chemical bond cleavage across the metal–ligand framework without requiring metal-centered redox changes.^5^ Likewise, multimetallic complexes can activate substrates through synergistic interactions between two or more metals, enabling modes of reactivity inaccessible to mononuclear systems.^6,7^ Renewed interest in multimetallic reactivity has stimulated the development of multinucleating ligands that enable the selective synthesis of well-defined multimetallic complexes and systematic investigation of their cooperative properties.^8^ These systems display distinct stoichiometric^9–17^ and catalytic^18–20^ reactivity and have been used to mimic surface facets relevant to heterogenous catalysis.^21–24^ The dinucleating ligands utilized in these multimetallic systems can broadly be divided into two classes: those that position metal centers within bonding distance, thereby enabling direct metal–metal interactions (class A), and those that prevent direct bonding interactions by geometrically enforcing metal–metal separation beyond the covalent bonding range (class B).^8^ Previous work from our group has focussed on a class A dinucleating ligands based on the widely employed 1,8-naphthyridine scaffold, a privileged platform for the construction of bimetallic complexes owing to its ability to support closely spaced metal centers (Scheme 1, top-left). In particular, our work has focused on the ^R^PNNP ligand platform (Scheme 1, top-right), which combines reversible dearomatization of the 1,8-naphthyridine backbone to enable metal–ligand cooperativity^25,26^ while the bimetallic core enables metal–metal cooperativity.^27,28^ However, the geometry imposed on the metals by the 1,8-naphthyridine backbone allows for the formation of stabilizing 3c-2e bonds (Scheme 1, top-middle), which can effectively tame otherwise highly reactive organometallic fragments, including copper-carbenes,^29^ -boryls^30^ and -hydrides,^31^ leading to diminished reactivity. While such stabilization can be beneficial in isolating well-defined intermediates, or enable particular pathways,^32^ it can also suppress the intrinsic reactivity required for bond activation and catalytic turnover.

**Scheme 1:**
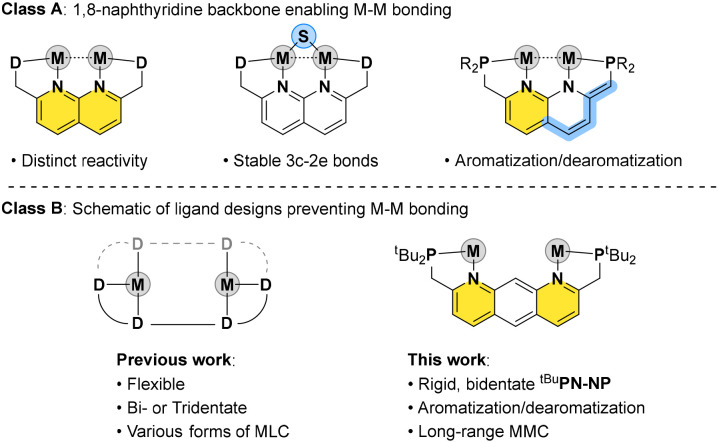
Top: Graphical representations of a bimetallic complex of a 1,8-naphthyridine-based class A type ligand, their 3c-2e bonding, and the ^R^PNNP scaffold. Bottom: Distinction between reported class B ligand designs and the presented 1,8-diazaanthracene based ^*t*Bu^PN-NP.

We hypothesized that a class B analogue of ^*t*Bu^PNNP ligand can circumvent the formation of these stabilizing 3c-2e bonds and generate more reactive metal–ligand fragments, enabling both MLC and long-range MMC. While class B ligands capable of supporting MLC and long range MMC have been reported, these differ in key structural aspects and coordination topology from the rigid dinucleating ^R^PNNP framework (*e.g.* with flexible backbones,^10,12^ or higher denticity coordination motifs,^33^Scheme 1, bottom-left). To address this, we designed a 1,8-diazaanthracene based ^*t*Bu^PN-NP ligand that features two spatially separated PN coordination pockets, while retaining the acidic methylene linkers within the P–N binding pockets. This ligand therefore provides a new platform to investigate long-range MMC in combination with aromatization-dearomatization-based MLC (Scheme 1, bottom-right).

Inspired by the synthesis of the previously reported ^*t*Bu^PNP^34^ and ^*t*Bu^PNNP^26^ ligands, we envisioned that double deprotonation of 2,7-dimethyl-1,8-diazaanthracene (DMDA), followed by the reaction with a corresponding chlorodialkylphosphine, would afford the desired ligand. While the synthesis of DMDA has been reported as a five-step procedure starting from *m*-xylene,^35^ we modified several reaction steps to improve yields or reproducibility (see S2.1–S2.5 for more detail). As anticipated, double deprotonation of DMDA using *n*-BuLi in THF and a subsequent reaction with two equiv. ^*t*^Bu_2_PCl afforded ^*t*Bu^PN-NP as a brown solid in 72% yield (Scheme 2b). The ^1^H, ^13^C and ^31^P{^1^H} NMR spectra of ^*t*Bu^PN-NP in CD_2_Cl_2_ at room temperature show the expected number of resonances for a *C*_2v_ symmetric species. The ^31^P{^1^H} NMR spectrum shows a single resonance at 33.5 ppm, similar to the ^31^P resonances found for the related ^*t*Bu^PNP (*δ* = 35.2 ppm in C_6_D_6_) and ^*t*Bu^PNNP (*δ* = 35.8 ppm in CD_2_Cl_2_) ligands.^26,34^ Reacting ^*t*Bu^PN-NP with two equiv. of Cu(MeCN)_4_(PF_6_) in CH_2_Cl_2_ results in selective formation of dicopper(i) complex 1, which was isolated in 98% yield. (Scheme 2b). Besides the expected small downfield shift of the ^1^H resonances of the heterocyclic scaffold, no significant changes are observed in the ^1^H NMR spectrum in comparison with the ^*t*Bu^PN-NP ligand. In contrast, a clear upfield shift is observed in the ^31^P{^1^H} NMR spectrum of 1 in CD_2_Cl_2_ (*δ* = 30.3 ppm) compared to ^*t*Bu^PN-NP (*δ* = 33.5 ppm in CD_2_Cl_2_), accompanied by the characteristic linebroadening arising from quadrupolar relaxation of the ^63^Cu and ^65^Cu nuclei (both *S* = 3/2).^36^

**Scheme 2:**
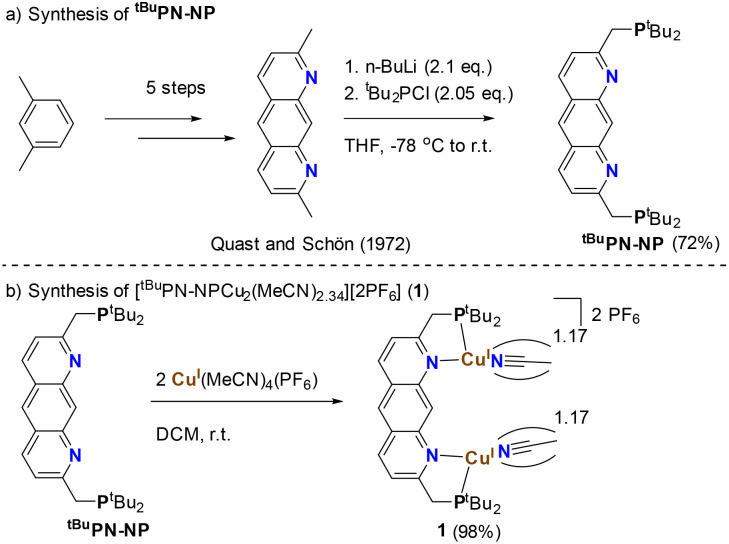
(a) Multistep synthesis of ^*t*Bu^PN-NP from *m*-xylene. (b) Synthesis of [Cu_2_(MeCN)_2_PN-NP][2PF_6_] (1).

Crystals suitable for single-crystal X-ray diffraction analysis were obtained from a MeCN/Et_2_O/pentane solution of 1. The solid state structure shows both copper atoms coordinated to the PN pockets and separated by 5.1013(10) Å (Fig. 1a). Whereas the NMR data indicate the formation of a symmetrical complex in solution, the two copper centers are non-equivalent in the solid state. To accommodate both copper atoms within the ligand framework, one copper adopts a distorted trigonal geometry while the second coordinates two MeCN atoms in a tetrahedral environment.‡‡The two coppers in the molecular structure of (1) in the crystal are disordered with a major disorder form: Cu1 tetrahedral, Cu2 trigonal and a minor disorder form: Cu1 trigonal, Cu2 tetrahedral. This is in contrast with ^1^H NMR analysis in CD_2_Cl_2_, which places a total of 2.34 MeCN molecules per complex. This assignment of ∼2 coordinated MeCN molecules per complex is further supported by elemental analysis, which showed good agreement between the calculated and experimental values for [Cu_2_(MeCN)_2_PN-NP][2PF_6_]. As the crystals were grown from MeCN solution, the coordination of three MeCN ligands is not surprising. In a CD_2_Cl_2_ solution, the species containing two trigonal copper centers is proposed to be dominant and to exist in rapid exchange with the structure shown in Fig. 1a, explaining the total of 2.34 MeCN molecules in 1. We observed similar fluxional behavior in structurally related dicopper–acetonitrile complexes supported by the ^R^PNNP ligand that binds two metals within bonding range.^29^

**Fig. 1:**
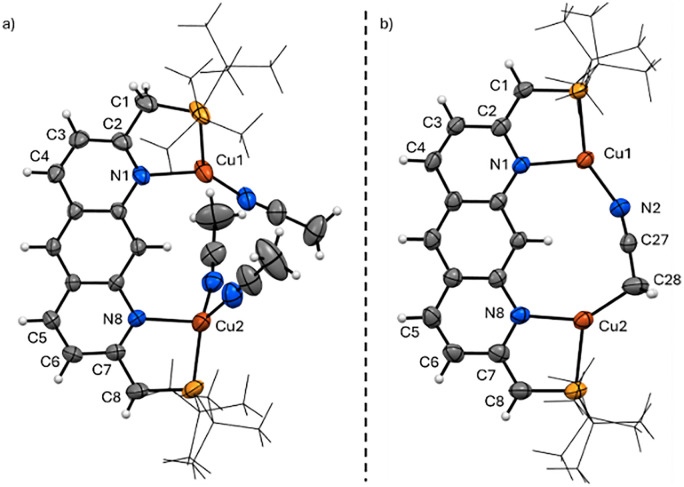
Molecular structures of [Cu_2_(MeCN)_3_^*t*Bu^PN-NP][2PF_6_] (1, a) and [Cu_2_(µ-CH_2_CN)PN-NP**][K(18-crown-6)(THF)_2_] (3, b) in the crystal (50% displacement ellipsoids, *t*Bu groups shown in wireframe). Counterions (2 PF_6_ for 1; K(18-c-6) for 3) and solvent molecules are omitted for clarity. Only the major disorder form of the coordinated acetonitrile molecules is shown. Selected distances (Å) for 1: Cu1⋯Cu2: 5.1013(10). Selected bond lengths for 3 (Å): Cu1⋯Cu2: 5.1178(9), Cu2–C28: 1.989(4), C27–C28: 1.386(5), C27–N2: 1.166(5).

To probe the ability of the 1,8-diazaanthracene backbone to dearomatize upon deprotonation of the methylene linkers, we reacted a suspension of [Cu_2_(MeCN)_2_PN-NP][2PF_6_] (1) in THF with one equivalent of the strong base potassium hexamethyldisilazide (KHMDS). This resulted in a rapid color change from yellow to purple. ^1^H NMR analysis of the crude reaction mixture indicated the formation of a mixture of species. Only upon the addition of a second equivalent of KHMDS did the reaction converge to two species, 2^a^* and 2^b^*(Scheme 3), which are formed in a 83 : 17 ratio. For both species, a loss of *C*_*2*v_ symmetry is observed, evident by an increase from four to six signals in the aromatic region of the ^1^H NMR for either of the two species. Deprotonation of one of the methylene linkers is evident by a diagnostic splitting of the four methylene protons in 1 (*δ*_H_ = 3.58 ppm, 4H) to a methine (*δ*_H_ = 4.32, 4.53 ppm, 1H, for 2^a^* and 2^b^* respectively) and a methylene signal (*δ*_H_ = 2.63, 2.57 ppm, 2 H, for 2^a^* and 2^b^* respectively). This is further corroborated by the ^31^P{^1^H} NMR spectrum in THF-*d*_8_ which shows two ^31^P resonances for either species, as the ^31^P resonance of the CH_2_–P^*t*^Bu_2_ moiety (*δ*_P_ = 20.57, 31.21 ppm, for 2^a^* and 2^b^* respectively) differs from the =CHP^*t*^Bu_2_ moiety (*δ*_P_ = 15.57, 2.00 ppm for 2^a^* and 2^b^* respectively). Overall, the loss of *C*_*2*v_ symmetry, accompanied by the diagnostic methyne is consistent with a partially dearomatized 1,8-diazaanthracene backbone for both species in solution. Interestingly, the ^1^H resonance formerly associated with MeCN in 1 (*δ*_H_ = 2.44), shows a large upfield shift in 2^a^* and 2^b^* (*δ*_H_ = 1.63, 2.03 ppm, respectively) and integrates for 2H. This suggests that the second equivalent of KHMDS deprotonates the end-on coordinated MeCN in 1 and forms a anionic cyanoalkyl (^−^CH_2_CN) ligand. This proposal is further supported by the observation of a distinct resonance at *δ*_c_ = −9.67 ppm in the ^13^C{^1^H} NMR spectrum. This is characteristic of aliphatic organocopper species,^37,38^ and the observed splitting pattern is consistent with the expected three-bond coupling to the adjacent phosphine donor (*J*_C–Cu–P_ = 35.8 Hz). The ATR-IR spectrum of 2 displays a weak absorption band at *ν* = 2194 cm^−1^, consistent with the C≡N stretching vibration of copper-cyanomethyl, which is shifted to lower wavenumbers in comparison to the nitrile stretching band in 1 (*ν*(C≡N) = 2282 cm^−1^). Based on ^1^H, ^13^C{^1^H} NMR and ATR-IR we propose the formation of a bridged dicopper species, [Cu_2_(μ-CH_2_CN)^*t*Bu^PN-NP*], which can exist as two isomers (2^a^* and 2^b^*, Scheme 3) that differ in the orientation of the cyanomethyl ligand due to partial dearomatization of the ^*t*Bu^PN-NP ligand. Consistent with this proposal, the addition of one additional equivalent of KHMDS and 18-crown-6 to this mixture of isomers results in an immediate color change from purple to pink and clean formation of 3. ^1^H NMR analysis of 3 in THF-*d*_8_ shows two sets of methine resonances at 3.45 and 3.30 ppm, and a further upfield shift of the aromatic resonances, indicative of double deprotonation and concomitant dearomatization of the ligand. Both ^1^H and ^31^P{^1^H} NMR indicate the presence of one nonsymmetric product, suggesting that the non-symmetric bridging cyanomethyl ligand remains intact upon double dearomatization. Layering a THF solution of 3 with pentane afforded red crystals, suitable for single-crystal X-ray diffraction analysis. The solid-state structure (Fig. 1b) confirms dearomatization on both sides of the 1,8-diazaanthracene, as evidenced by the shortening of the C–C bonds between the methine and diazaanthracene carbon atoms by 0.13 Å (C1–C2 and C7–C8, see Fig. S21) compared the bond lengths in the percursor 1. The solid state structure further confirms the presence of a bridging, cyanomethyl ligand between both copper centers. In 3, the Cu···Cu separation is slightly increased relative to 1 (5.1178(9) *vs.* 5.1013(10) Å, respectively). While the X-ray crystal structure shows significant shortening of the carbon–carbon bond of μ-CH_2_CN in 3 compared to the MeCN molecules in 1, the C–N bond distance does not change significantly, indicating that the triple bond remains intact (see Fig. S21). This M_2_(µ-η^1^(C):η^1^(N)-CH_2_CN) motif is rare: only thirteen related structures reported in the Cambridge Structural Database^39^ (accessed 29-05-2026), all corresponding to aggregates of mononuclear complexes, and none containing copper. This illustrates the ability of the ^*t*Bu^PN-NP ligand framework to support a unique bimetallic core with large metal–metal separation, thereby enabling stabilization of reactive fragments such as ^−^CH_2_C≡N.

**Scheme 3:**
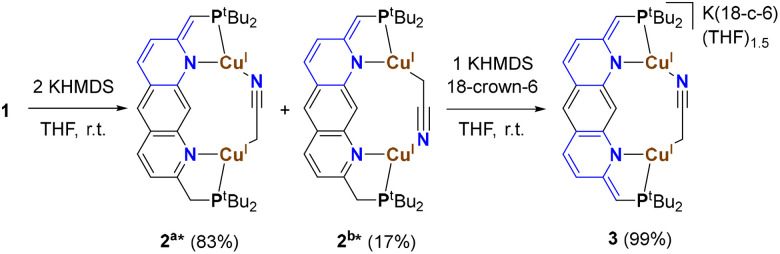
Synthesis of [Cu_2_(μ-MeCN)^*t*Bu^PN-NP*] (2*), which consists of two isomers (2^a^* and 2^b^*) (left) and synthesis of [Cu_2_(μ-MeCN)^*t*Bu^PN-NP**][K(18-crown-6)(THF)_1.5_] (3) (right).

Upon stepwise dearomatization of the diazaanthracene backbone during the conversation of 1 to 2 and subsequently to 3, the color of the complexes changed from brown to intense purple and finally to pink, respectively. These optical properties motivated further investigation of the electronic structure using UV-vis spectroscopy to determine whether the observed color changes are due to ligand-centered or metal-centered transitions. To distinguish between these possibilities, the optical properties of the free ligand were first investigated. Reacting a brown suspension of ^*t*Bu^PN-NP in THF with 1 equivalent of KHMDS and 18-crown-6 at room temperature, resulted in immediate formation of a intense blue solution, associated with the formation of ^*t*Bu^PN-NP*(Scheme 4). The resonances observed in ^1^H and ^31^P{^1^H} NMR spectra are consistent with an nonsymmetric diazaanthracene backbone and partial dearomatization (See Fig. S14). The intense blue color of ^*t*Bu^PN-NP* is reflected by multiple strong absorbance bands around 600 nm, which are absent in the UV-vis spectrum of ^*t*Bu^PN-NP (Fig. 2, top). ^*t*Bu^PN-NP* is best prepared *in situ* due to the gradual decomposition of the material in the solid state. ^1^H NMR analysis of a reaction mixture of ^*t*Bu^PN-NP with 2 equivalents of KHMDS and 18-crown-6 showed a mixture of ^*t*Bu^PN-NP* and the doubly dearomatized analogue (^*t*Bu^PN-NP**). However, the addition of an excess of KHMDS (17 equiv.), or two equivalents of the stronger alkali base BnK, resulted in full conversion of ^*t*Bu^PN-NP to ^*t*Bu^PN-NP**(Scheme 4). This observation indicates a significantly higher p*K*_a_ for the second deprotonation step, and the pronounced basicity of ^*t*Bu^PN-NP** is reflected in its decomposition upon solvent removal.

**Scheme 4:**
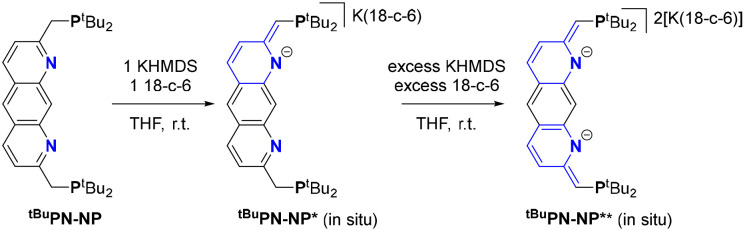
Synthesis of K(18-c-6)^*t*Bu^PN-NP* (top) and of K_2_^*t*Bu^PN-NP** (bottom) through deprotonation with KHMDS.

**Fig. 2:**
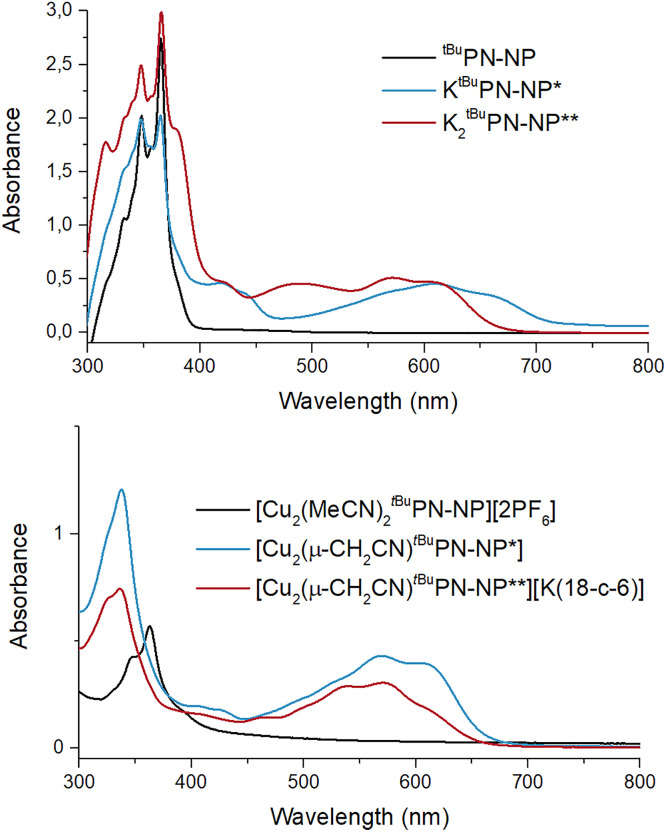
Top: UV-vis spectra of 0.16 mM solutions of ^*t*Bu^PN-NP, K^*t*Bu^PN-NP*, K_2_^*t*Bu^PN-NP** in THF, generated by the *in situ* deprotonation of ^*t*Bu^PN-NP with KHMDS/18-c-6 (1 equiv.) and BnK (2 equiv.), respectively. Bottom: UV-vis spectra of 0.01 mM solutions of [Cu_2_(MeCN)_2_^*t*Bu^PN-NP][2PF_6_] (1), [Cu_2_(μ-MeCN)^*t*Bu^PN-NP*] (2^a^* and 2^b^*), [Cu_2_(μ-MeCN)^*t*Bu^PN-NP**][K(18-crown-6)] (3) in THF.

THF solutions of ^*t*Bu^PNNP** exhibit an intense red color, corresponding to a new absorption band at 480 nm. This is accompanied by a red-shift of the *λ*_max_ of the partially dearomatized compound from 607 nm to 570 nm in the UV-vis spectrum (Fig. 2, top). Similar to the non-coordinated ligand system, the stepwise deprotonation from 1 to 2, and subsequently 3 is accompanied by pronounced color changes from brown-yellow to deep purple and finally to intense pink, suggesting a similar evolution in the absorption properties. To investigate these changes, UV-vis spectra of the dicopper complexes 1–3, which span the three possible protonation states of the dinucleating ligand, were recorded in THF solution. Comparing the UV-vis spectra of ^*t*Bu^PN-NP to 1 only shows a slight red-shift of the characteristic 1,8-diazaanthracene absorption bands in the 300-400 nm region,^35^ while no new absorbance bands appear (Fig. 2, black traces). This indicates that coordination of the copper centers in 1 only has a minor influence on the electronic transitions of the ligand framework. Upon partial dearomatization of the 1,8- diazaanthracene backbone in ^*t*Bu^PN-NP*, new low-energy absorption bands emerge in the UV-vis spectra with *λ*_max_ at 610 nm, which are also found in 2, but slightly blueshifted to 570 nm, respectively (Fig. 2, blue traces). Double dearomatization of the free ligand to form ^*t*Bu^PN-NP** results in a further red shift of this feature to 570 nm in comparison with ^*t*Bu^PN-NP*. Although less pronounced, similar changes in absorption features are observed upon conversion from 2 to 3, showing a subtle red shift of approximately 50 nm (Fig. 2, red traces) Together, these observations indicate that the intense visible absorption bands in the dicopper complexes are primarily ligand-centered π–π* transitions.

In conclusion, the 1,8-diazaanthracene-supported dinucleating ligand framework reported here enables the combined study of long-range metal–metal cooperativity and metal–ligand cooperativity in small-molecule activation. Coordination of two copper centres within the ^*t*Bu^PN-NP scaffold gives a rigid bimetallic structure with a well-defined, large Cu···Cu separation. This platform was demonstrated to enable the generation of a nitrile-derived, bridged cyanomethyl fragment, highlighting how remote metal centres can act cooperatively to generate a reactive intermediate.

## Conflicts of interest

There are no conflicts to declare.

## Supplementary Material

CC-062-D6CC03416K-s001

CC-062-D6CC03416K-s002

## Data Availability

The spectroscopic data related to the work described in this paper is available free of charge in the Yoda data repository at https://doi.org/10.24416/UU01-HIG7QO. Supplementary information (SI) is available. See DOI: https://doi.org/10.1039/d6cc03416k. CCDC 2557890 and 2557891 contain the supplementary crystallographic data for this paper.^40*a*,*b*^
