# Supplementary material for: Long-range metal–metal cooperative nitrile activation in 1,8-diazaanthracene-supported dicopper complexes
Source: Chem Commun (Camb). 2026 Jun 24;62(59):14836–40. doi: 10.1039/d6cc03416k (PMC13359027; doi:10.1039/d6cc03416k)
Supplement: CC-062-D6CC03416K-s001 [file CC-062-D6CC03416K-s001.pdf]

Supplementary Information (SI) for ChemComm.
This journal is © The Royal Society of Chemistry 2026

Supporting Information

Long-range Metal-Metal Cooperative Nitrile Activation in 1,8-Diazaanthracene-supported Dicopper Complexes

Arie J.H. Multem[#a], Vishal Chugh[#a], Annemijn M. van Kooten[a], Mick J. Zomer,[a] Martin Lutz[b], Daniël L. J. Broere*[a]

a: Organic Chemistry and Catalysis, Institute for Sustainable and Circular Chemistry, Faculty of Science, Utrecht University, Universiteitsweg 99, 3584 CG Utrecht, The Netherlands

b: Structural Biochemistry Bijvoet Centre for Biomolecular Research, Faculty of Science, Utrecht University, Universiteitsweg 99, 3584 CG Utrecht, The Netherlands

*Corresponding Author: d.l.j.broere@uu.nl

Table of contents

S1. General considerations

S2. Synthesis and characterization

- S2.1 Synthesis of 1,5-dimethyl-2,4-dinitrobenzene
- S2.2 Synthesis of (1*E*,1'*E*)-2,2'-(4,6-dinitro-1,3-phenylene)bis(N,N-dimethylethen-1-amine)
- S2.3 Synthesis of 4,6-dinitroisophthalaldehyde
- S2.4 Synthesis of 4,6-diamineisopthalaldehyde
- S2.5 Synthesis of 2,7-dimethyl-1,8-diazaanthracene (**DMDA**)
- S2.6 Synthesis of $^{\boldsymbol{tBu}}$PN-NP
- S2.7 [Cu_2(MeCN)$_{\mathbf{2}}$$^{\boldsymbol{tBu}}$PN-NP][2$PF_6$](**1**)
- S2.8 [Cu_2(μ-MeCN)^{tBu}PN-NP*] (**2^{a}*** and **2^{b}***):
- S2.9 [Cu_2(μ-MeCN)^{tBu}PN-NP**][K(18-crown-6)] (**3**):
- S2.10 *In situ* generation of ^{tBu}PN-NP*
- S2.11 *In situ* generation of ^{tBu}PN-NP**

S3. NMR spectra

- S3.1 ^{tBu}PN-NP
- S3.2 [Cu_2(MeCN)$_{\mathbf{2}}$$^{\boldsymbol{tBu}}$PN-NP][2$PF_6$](**1**)
- S3.3 [Cu_2(μ-MeCN)^{tBu}PN-NP*] (**2^{a}*** and **2^{b}***)
- S3.4 [Cu_2(μ-MeCN)^{tBu}PN-NP**][K(18-crown-6)] (**3**)
- S3.5 ^{tBu}PN-NP*
- S3.6 ^{tBu}PN-NP**

S4. IR spectra

- S4.1 $^{\boldsymbol{tBu}}$PN-NP
- S4.2 [Cu_2(MeCN)$_{\mathbf{2}}$$^{\boldsymbol{tBu}}$PN-NP][2$PF_6$](**1**)
- S4.3 [Cu_2(μ-MeCN)^{tBu}PN-NP*] (**2^{a}*** and **2^{b}***)
- S4.4 [Cu_2(μ-MeCN)^{tBu}PN-NP**][K(18-crown-6)] (**3**)

S5 HRMS spectrum of ^{tBu}PN-NP

S6. X-ray crystal structures:

- S6.1 X-ray crystal structure determination of (**1**)
- S6.2 X-ray crystal structure determination of (**3**)
- S6.3 Metric parameters of (**1**) and (**3**)

S7. References

S1. General considerations

All experimental manipulations were carried out under an inert nitrogen atmosphere using either standard Schlenk techniques or within a nitrogen-filled M. Braun glovebox unless stated otherwise. The internal glovebox temperature was typically maintained near ambient conditions (approximately 298–303 K). Glassware was dried in an oven at 130 °C for a minimum of 12 h or heated under dynamic vacuum prior to use.

Hexane, Et_2O, toluene and DCM were collected from an M. Braun MB-SPS-800 solvent purification system, degassed and stored over molecular sieves (4 Å; 3 Å for CH_2Cl_2). Tetrahydrofuran was purified either by distillation from sodium/benzophenone ketyl or sodium-dispersed media, followed by vacuum transfer and further stored over 4 Å molecular sieves. Solvent quality was verified using a sodium benzophenone ketyl indicator solution and/or quantification of residual water content by Karl Fischer titration.

Deuterated solvents (including THF-d_8 and CD_2Cl_2) were obtained from Cambridge Isotope Laboratories or ABCR, degassed by freeze–pump–thaw cycles, and stored over molecular sieves (4 Å; 3 Å for CD_2Cl_2). All commercial reagents were purchased from standard suppliers (e.g., BLD Pharma, Sigma-Aldrich, ABCR) and used as received. Liquid reagents were degassed by freeze–pump–thaw cycling or N_2 sparging, while solid reagents were degassed under dynamic vacuum prior to use.

NMR data was recorded on an VNMRS-400 Varian 400 MHz (9.4 T) NMR system equipped with an OneNMR probe (with quartz liner) and Optima Tune system and a Performa IV PFG amplifier capable of generating a 65 G/cm gradient or a Jeol JNM-ECZL G 400 MHz (9.4 T) NMR system equipped with an autotunable ROYALPROBE HFX (with quartz liner) and a gradient amplifier capable of generating a 90 G/cm gradient. Chemical shifts (δ, ppm) for ^{1}H and ^{13}C NMR were referenced to residual solvent resonances. ^{31}P and ^{19}F NMR spectra were referenced using the absolute frequency method relative to a properly referenced ^{1}H spectrum of the same sample. Signal assignments were based on chemical shift, integration, multiplicity, and supported by 2D NMR experiments (COSY, HMQC, HMBC). For ^{1}H NMR spectra acquired in non-deuterated solvents, solvent suppression techniques (PRESAT or WET) were employed. Air- and moisture-sensitive samples were measured in sealed J. Young NMR tubes under N_2 atmosphere. ATR-FTIR spectra were collected on a PerkinElmer Frontier spectrometer equipped with a PerkinElmer Universal ATR Sampling Accessory with a diamond/ZnSe plate and a LiTaO3 mid-IR detector. Samples sensitive to air were prepared by drop-casting solutions in THF onto the ATR crystal under a continuous nitrogen flow. High-Resolution Mass Spectrometry (HR-MS) measurements were performed using an Agilent Technologies 6560 IMS-QTOF mass spectrometer. Elemental analyses were performed by MEDAC Ltd., UK.

S2. Synthesis and characterization

S2.1 Synthesis of 1,5-dimethyl-2,4-dinitrobenzene

All manipulations were performed under aerobic conditions using technical grade solvents.

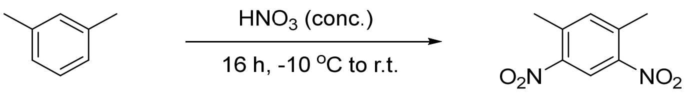

Following an adapted literature procedure[1], *m*-Xylene (100 mL, 86 g, 0.81 mol, 1 eq.) was added dropwise over 1.5 h to a stirred concentrated nitric acid (190 mL, 287 g, 4.55 mol, 5.6 eq.) at -10 °C, during which the reaction mixture turned dark red. After addition, the mixture was allowed to warm to room temperature, forming a clear orange solution. The mixture was stirred at room temperature for 16 h. Dropwise addition of the reaction mixture to a vigorously stirred ice/water mixture (2 L), resulting in the formation of a light yellow precipitate. The precipitate was collected by filtration and washed with water (1 L) and MeOH (3 x 500 mL). The residue was dried under a dynamic vacuum to give a light yellow crude powder. The crude powder was purified by repeated recrystallizations from hot methanol (0.2 g/mL) until pure by TLC analysis, R_F = 0.38 (EtOAc/heptane, 1:3) , to yield 1,5-dimethyl-2,4-dinitrobenzene as light yellow crystals (50.96 g, 227 mmol, 32%).

^{1}H NMR spectrum is consistent with the reported literature data.[1]

^{1}H NMR (400 MHz, $CDCl_3$, 298 K): *δ* 8.67 (s, 1H), 7.37 (s, 1H), 2.68 (s, 6H).

^{13}C{^{1}H} NMR (100 MHz, $CDCl_3$, 298 K): *δ* 146.8, 139.2, 137.5, 121.9, 20.7.

S2.2 Synthesis of (1*E*,1'*E*)-2,2'-(4,6-dinitro-1,3-phenylene)bis(N,N-dimethylethen-1-amine)

All manipulations were performed under aerobic conditions using technical grade solvents.

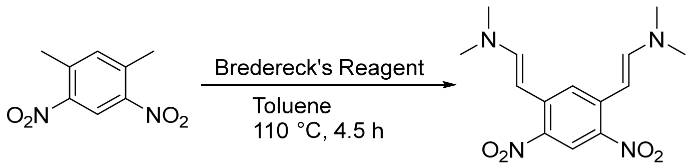

Bredereck's reagent (90% purity, 40.0 mL, 30.6 g, 176 mmol, 2.5 eq.) was added dropwise to a stirred solution of 1,5-dimethyl-2,4-dinitrobenzene (14.0 g, 71.2 mmol, 1 eq.) in toluene (150 mL), during which the reaction mixture turned dark blue and gradually dark red upon complete addition. The mixture was heated at 110 °C for 4.5 h. After cooling to ambient temperature, the mixture was concentrated under reduced pressure to approximately 40 mL. The resulting suspension was filtered, the residue was washed with pentane (3 × 50 mL) and dried under dynamic vacuum, to yield (1*E*,1'*E*)-2,2'-(4,6-dinitro-1,3-phenylene)bis(N,N-dimethylethen-1-amine) as dark purple crystals (21.0 g, 65.3 mmol, 92%).

^{1}H NMR spectrum is consistent with the reported literature data[2]

^{1}H NMR (400 MHz, $CDCl_3$, 298 K): *δ* 8.71 (s, 1H), 7.21 (s, 1H), 7.05 (d, *J* = 13.2 Hz, 2H), 6.00 (d, *J* = 13.2 Hz, 2H), 2.98 (s, 12H).

^{13}C{^{1}H} NMR (100 MHz, $CDCl_3$, 298 K): *δ* 146.7, 140.3, 138.2, 125.9, 116.2, 91.6

S2.3 Synthesis of 4,6-dinitroisophthalaldehyde

All manipulations were performed under aerobic conditions using technical grade solvents.

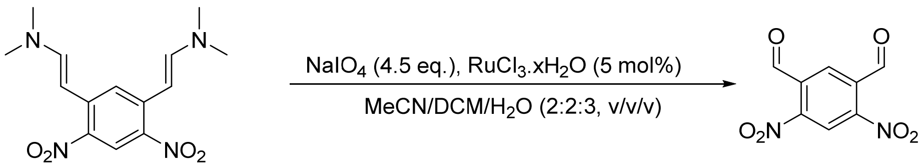

The compound can be prepared following an literature procedure,[3] however this resulted in inconsistent results with yield ranging from 5-99%. Hence we devised a more robust synthesis method that provided more consistent yields (44-48%, n = 4). $NaIO_4$ (66.9 g, 313 mmol, 4.5 eq.) and $RuCl_3$ x H_2O (710 mg, 5 mol%) in MeCN/H_2O (2:3, 400 mL MeCN, 600 mL H2O) was added to a mechanically stirred solution of (1*E*,1'*E*)-2,2'-(4,6-dinitro-1,3-phenylene)bis(N,N-dimethylethen-1-amine) (21.3 g, 69.5 mmol, 1 eq.) in DCM (400 mL). The suspension was stirred at r.t. for 20 h during which the reaction mixture turned from red to yellow-brown and showed significant formation of white solids. Full conversion of the starting material was confirmed by measuring an solvent-suppressed ^{1}H NMR spectrum of an aliquot of the DCM layer. The mixture was concentrated under reduced pressure to a final volume of 600 mL. The remaining suspension was filtered, the filter cake washed with EtOAc (2 x 100 mL) and conc. $NaHCO_3$ solution (400 mL) was added to the filtrate. The aqeous fraction was extracted with EtOAc (500 mL), the organic layers were combined, washed with brine, dried over $MgSO_4$, and the volatiles removed under reduced pressure. The residue was purified by column chromatography (EtOAc/heptane, 0 → 30% EtOAc) to yield 4,6-dinitroisophthalaldehyde as orange powder (7.45 gram, 33.2 mmol, 48%).

^{1}H NMR spectrum is consistent with the reported literature data[3]

^{1}H NMR (400 MHz, $CDCl_3$, 298 K): δ 10.49 (s, 2H), 8.87 (s, 1H), 8.46 (s, 1H).

^{13}C{^{1}H} NMR (100 MHz, $CDCl_3$, 298 K): δ 185.2, 150.7, 135.1, 132.6, 121.6.

S2.4 Synthesis of 4,6-diamineisopthalaldehyde

All manipulations were performed under aerobic conditions using technical grade solvents.

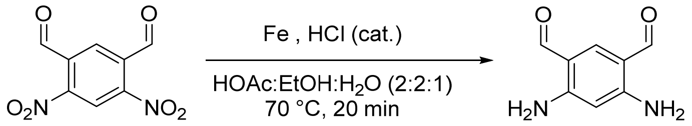

Following an adapted literature procedure[3], iron powder (14.9 g, 266 mmol, 8 eq.) was added portion wise to a stirred solution of 4,6-dinitroisophthalaldehyde (7.45 g, 33.2 mmol, 1 eq.) in AcOH/EtOH/H_2O (125 mL, 2:2:1) at 0 °C. After 10 min, concentrated HCl (0.5 mL) was added dropwise. The reaction mixture was heated to 70 °C for 20 min. After cooling to ambient temperature, the black suspension was filtered through a pad of celite and the filter cake was washed with hot EtOH (2 x 100 mL). Using an aqueous NaOH solution (1M, ~150 mL), the pH of the filtrate is adjusted to 8, resulting in the formation of a green precipitate. The green suspension was filtered through a pad of celite, the filter cake was washed with $CHCl_3$ (3 x 100 mL) and conc. $NaHCO_3$ solution (aq, 400 mL) was added. The organic fraction was extracted with $CHCl_3$ (3 x 200 mL), the organic layers combined, washed with brine, dried over $MgSO_4$ and the volatiles removed under reduced pressure. The dark orange residue

was dissolved in a minimal amount of DCM and charcoal (5 wt%) is added to remove residual iron particles. The suspension was filtered through a pad of celite, and the filtrate was concentrated under reduced pressure till dryness to obtain 4,6-diamineisopthalaldehyde as a yellow powder (3.45 gram, 21.0 mmol, 63 %).

^{1}H NMR spectrum is consistent with the reported literature data[3]

^{1}H NMR (400 MHz, $CDCl_3$, 298 K): δ 9.64 (s, 2H), 7.65 (s, 1H), 6.48 (bs, 4H), 5.65 (s, 1H).

^{13}C{^{1}H} NMR (100 MHz, $CDCl_3$, 298 K): δ 190.9, 154.5, 148.7, 113.1, 96.8

S2.5 Synthesis of 2,7-dimethyl-1,8-diazaanthracene (**DMDA**)

All manipulations were performed under aerobic conditions using technical grade solvents.

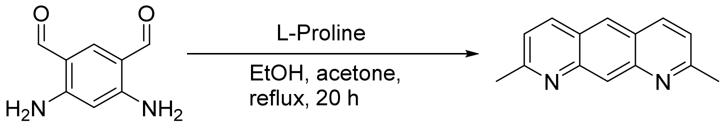

Following an adapted literature procedure[1], 4,6-diamineisopthalaldehyde (3.41 g, 20.8 mmol, 1 eq.) and L-proline (4.72 g, 41.0 mmol, 2 eq.) was dissolved in EtOH (200 mL) and acetone (120 mL). The reaction mixture was heated at reflux for 16 h while stirring. After cooling to room temperature, the mixture was concentrated under reduced pressure to give an orange solid, which was suspended in water (100 mL). The organic fraction was extracted with DCM (3x 100 mL), the organic layers were combined, washed with brine (100 mL), dried over $MgSO_4$, the volatiles removed under reduced pressure and purified by column chromatography (EtOAc/heptane, 1:1, Rf =) to yield 2,7-dimethyl-1,8-diazaanthracene (**DMDA**) as a yellow powder (2.8 gram, 65%).

^{1}H NMR spectrum is consistent with the reported literature data[1]

^{1}H NMR (400 MHz, $CDCl_3$, 298 K): δ 8.74 (s, 1H), 8.24 (s, 1H), 8.19 (d, *J* = 8.7 Hz, 2H), 7.28 (d, *J* = 8.7 Hz, 2H), 2.80 (s, 6H).

^{13}C{^{1}H} NMR (100 MHz, $CDCl_3$, 298 K): δ 161.0, 146.9, 136.3, 126.8, 126.5, 125.0, 122.4, 26.1.

S2.6 Synthesis of **^{tBu}PN-NP**

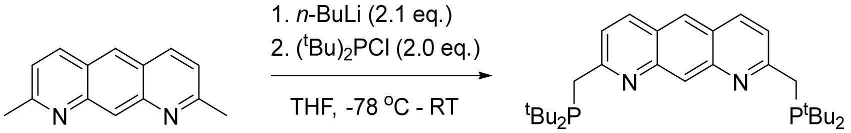

^{tBu}PN-NP

A 1.6 M solution of *n*-BuLi in hexanes (3.2 mL, 5.1 mmol, 2.1 eq.) was cannulated dropwise to a stirred brown suspension of 2,7-dimethyl-1,8-diazaanthracene (500 mg, 2.4 mmol, 1.0 eq.) in THF (20 mL) at -78 °C, during which the reaction mixture turned an intense purple. After addition, the reaction mixture was warmed to room temperature and a color change to reddish-brown was observed. The reaction mixture was cannulated dropwise minutes to a solution of di-tert-butylchlorophosphine (0.91 mL, 0.87 g, 4.8 mmol, 2.0 eq.) in THF (5 mL) at -78 °C, during which the reaction mixture turned an

intense purple. After addition, the mixture was allowed to warm to room temperature, and stirred for 16 h. The reaction was quenched by the dropwise addition of degassed water (10 mL) to give a brown suspension. The organic fraction was extracted with DCM (1x 25 mL, 2 x 10 mL), the organic layers were combined, dried over Na_2SO_4 and the volatiles removed under reduced pressure to give brown crude powder. The crude powder was washed with pentane (10 mL), diethyl ether (12 mL) and dried under dynamic vacuum to give **^{tBu}PN-NP** as a brown powder (858 mg, 1.73 mmol, 72%).

^{1}H NMR (400 MHz, THF-d_8, 298 K): δ 8.50 (s, 1H), 8.30 (s, 1H), 8.23 (dd, *J* = 9.0, 0.9 Hz, 2H), 7.56 (d, *J* = 8.7 Hz, 2H), 3.30 (d, *J* = 3.2 Hz, 4H), 1.19 (d, *J* = 10.8 Hz, 36H) ppm.

^{13}C{^{1}H} NMR (101 MHz, THF-d_8, 298 K): δ 165.05 (d, *J* = 14.4 Hz), 147.8, 136.3, 127.7, 127.2, 126.2, 123.3 (d, *J* = 8.6 Hz), 34.6 (d, *J* = 27.3 Hz), 32.9 (d, *J* = 24.1 Hz), 30.3 (d, *J* = 13.8 Hz) ppm.

^{31}P{^{1}H} NMR (162 MHz, THF-d_8, 298 K): δ 33.54 (s) ppm.

IR-ATR (cm^{-1}): 2940 (m), 1589 (m), 1430 (m), 1366 (m), 885 (s), 814 (m).

HR-MS (ESI+, m/z): calc. for $C_{30}H_{47}N_2P_2$ $[M-H]^+$ 497.321449. Found 497.3219.

S2.7 Synthesis of [$Cu_2(MeCN)_2$^{tBu}PN-NP][2PF_6](**1**)

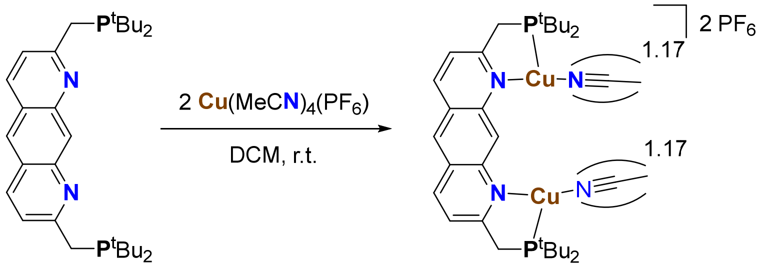

^{tBu}PN-NP **1**

A brown solution of **^{tBu}PN-NP** (198 mg, 0.4 mmol, 1 eq.) in DCM (10 mL) was added dropwise to a stirred, clear solution of $[Cu(MeCN)_4]PF_6$ (305 mg, 0.8 mmol, 2 eq.) in DCM (24 mL), during which the reaction mixture turned orange-brown. The reaction mixture was stirred at room temperature for 2 h. The volatiles were removed under reduced pressure to give orange-brown powder. The powder was washed with pentane (5 mL), extracted with DCM, concentrated under a dynamic vacuum and stripped with pentane (2 x 5 mL) to yield [$Cu_2(MeCN)_{2.34}$^{tBu}PN-NP][2PF_6](**1**) as orange-brown powder (404 mg, 98 %). Single crystals of **1** suitable for X-ray diffraction were grown by layering a MeCN solution of **1** with Et_2O.

*The number of coordinated MeCN molecules was quantified by ^{1}H qNMR spectroscopy using the relative integration of the MeCN ^{1}H resonance with respect to the ligand ^{1}H resonances.

^{1}H NMR (400 MHz, CD_2Cl_2, 298 K): δ 8.91 (s, 1H, 7), 8.68 (s, 1H, 6), 8.62 (d, *J* = 8.7 Hz, 2H, 5), 7.68 (d, *J* = 8.7 Hz, 2H, 4), 3.58 (d, *J* = 8.0 Hz, 4H, 8), 2.44 (s, 7H, 13), 1.30 (d, *J* = 14.5 Hz, 36H, 11) ppm.

^{13}C{^{1}H} APT NMR (101 MHz, CD_2Cl_2, 298 K): δ 166.53 (3), 145.33 (1), 140.86 (5), 129.98 (6), 126.69 (2), 125.33 (7), 124.25 (d, *J* = 3.8 Hz, 4), 121.65 (12), 33.14 (d, *J* = 11.4 Hz, 8), 32.79 (d, *J* = 16.2 Hz, 10), 29.59 (d, *J* = 7.9 Hz, 11), 3.24 (13) ppm.

^{31}P{^{1}H} NMR (162 MHz, CD_2Cl_2, 298 K): δ 30.27 (s), -143.9 (sept, $^{1}J_{P-F}$ = 710.9 Hz) ppm.

^{19}F NMR(376 MHz, $CDCl_3$, 298 K): -72.73 (d, $^{1}J_{F\text{-}P}$ = 710.9 Hz)

IR-ATR (cm^{-1}): 2946 (m), 2282 (w), 1622 (w), 1587 (w), 1394 (w), 1372 (m), 1295 (w), 1295 (w), 1268 (w), 1180 (w), 1021 (w), 829 (s), 732 (s), 701 (m), 617 (w).

Anal. Calcd. For $C_{34}H_{52}Cu_2F_{12}N_4P_4$: C, 41.01; H, 5.26; N, 5.63 %. Found C, 40.76; H, 5.53; N, 5.60 %. The calculated values are provided for 2 coordinated MeCN molecules.

S2.8 Synthesis of [$Cu_2(\mu\text{-}CH_2CN)^{tBu}$PN-NP*] (**2^{a}*** and **2^{b}***)

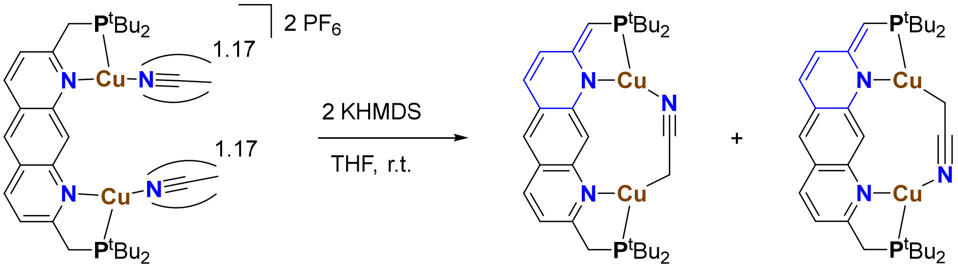

1 **2^{a*}** **2^{b*}**

A solution of KHMDS (20.0 mg, 0.1 mmol, 2 eq.) in THF (1 mL) was added dropwise to a stirred, orange-brown suspension of **1** (50 mg, 0.05 mmol, 1 eq.) in THF (1 mL), during which the reaction mixture turned purple, then pink and back to purple. The reaction mixture was stirred at room temperature for 1 h. The volatiles were removed under reduced pressure to give a purple powder. The powder was extracted with benzene (~ 2 mL), extracts were filtered, after which all volatiles were removed under a dynamic vacuum to yield [$Cu_2(\mu\text{-}CH_2CN)^{tBu}$PN-NP*] (**2^{a}*** and **2^{b}***) as a purple crystalline powder (32 mg, >99% combined yield).

Complex **2^{a}***:*
^{1}H NMR (400 MHz, C_6D_6, 298 K): δ 8.46 (s, 1H), 7.41 (d, *J* = 8.0 Hz, 1H), 7.01 (s, 1H), 6.83 – 6.64 (m, 3H), 6.33 (d, *J* = 8.0 Hz, 1H), 4.32 (d, *J* = 1.9 Hz, 1H), 2.63 (d, *J* = 6.3 Hz, 2H), 1.63 (d, *J* = 1.3 Hz, 2H), 1.28 (d, *J* = 13.4 Hz, 18H), 0.83 (d, *J* = 12.9 Hz, 18H) ppm.

^{13}C APT NMR (101 MHz, C_6D_6, 298 K) 164.81 (d, *J* = 13.9 Hz), 159.69 (d, *J* = 5.6 Hz), 154.81 (d, *J* = 5.7 Hz), 150.09 (d, *J* = 4.9 Hz), 138.74, 137.13 (d, *J* = 1.7 Hz), 129.39 (d, *J* = 9.2 Hz), 128.98, 114.75 (d, *J* = 3.1 Hz), 112.89, 82.03 (d, *J* = 42.5 Hz), 32.91 (d, *J* = 15.1 Hz), 31.92 (d, *J* = 1.7 Hz), 31.68 (d, *J* = 9.5 Hz), 29.93 (d, *J* = 9.1 Hz), 29.27 (d, *J* = 9.0 Hz), -9.67 (d, *J* = 35.8 Hz).

^{31}P{^{1}H} NMR (162 MHz, C_6D_6, 298 K): δ 20.57 (bs, 22), 15.57 (bs, 21).

Complex **2^{b}***:*
^{1}H NMR (400 MHz, C_6D_6, 298 K): δ 8.40 (s, 1H), 7.38 (d, *J* = 8.5 Hz, 2H), 6.97 (s, 1H), 6.79 (s, 2H), 6.72 (s, 2H), 6.25 (d, *J* = 8.0 Hz, 1H), 4.53 (d, *J* = 3.0 Hz, 1H), 2.57 (d, *J* = 7.9 Hz, 2H), 2.03 (s, 2H), 1.28 (d, *J* = 12.3 Hz, 18H), 0.77 (d, *J* = 13.3 Hz, 18H) ppm.

^{13}C{^{1}H} APT NMR (101 MHz, C_6D_6, 298 K): The concentration of **2^{b}*** is too low to discern ^{13}C resonances.

^{31}P{^{1}H} NMR (162 MHz, C_6D_6, 298 K): δ 31.21 (bs), 2.00 (bs).

IR-ATR (cm^{-1}): 2948 (m), 2194 (w), 1641 (m), 1617 (m), 1578 (m), 1464 (s), 1393 (m), 1363 (m), 1308 (m), 1159 (m), 1112 (m), 1018 (m), 882 (w), 841 (s), 814 (s), 620 (w).

Anal. Calcd. For $C_{32}H_{47}Cu_2N_3P_2$: C, 57.99; H, 7.15; N, 6.34 %. Found C, 57.84; H, 6.93; N, 5.32 %. The reactive nature of this compound precluded obtaining a satisfactory result for the elemental analysis. Instead, the purity of **2** was quantified by ^{1}H qNMR spectroscopy using trimethoxybenzene as internal standard as 99.1%.

S2.9 Synthesis of [Cu_2(μ-CH_2CN)^{tBu}PN-NP**][K(18-crown-6)(THF)$_{1.5}$] (**3**)

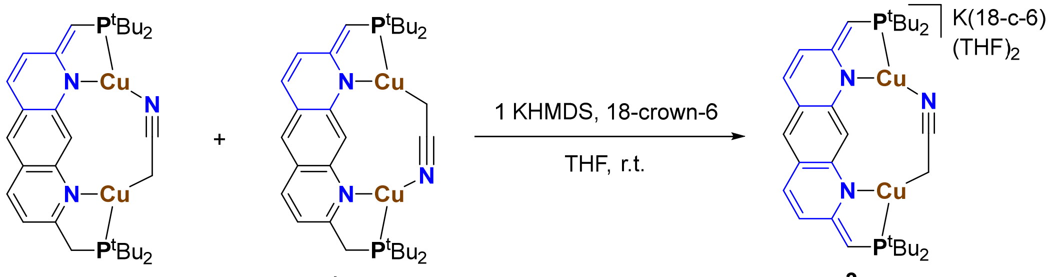

2^{a*} (83%) **2^{b*}** (17%) **3**

A solution of KHMDS (6.2 mg, 0.031 mmol, 1 eq.) and 18-crown-6 (8.2 mg, 0.031 mmol, 1 eq.) in THF (1 mL) was added dropwise to a stirred purple solution of **2** (20.7 mg, 0.031 mmol, 1 eq.) in THF (1 mL) The resulting pink mixture was stirred at room temperature for 1 h, after which volatiles were removed under reduced pressure. The residue was stripped with pentane (1 x 2 mL) to yield [Cu_2(μ-CH_2CN)^{tBu}PN-NP**][K(18-crown-6)(THF)$_{1.5}$] (**3**) as a pink powder (29 mg, 96%). Single crystals of **3** suitable for X-ray diffraction were grown by vapor diffusion pentane into a concentrated solution of **3** in THF.

*The number of coordinated THF molecules was quantified by ^{1}H qNMR spectroscopy using the relative integration of the THF ^{1}H resonances with respect to the ligand ^{1}H resonances.

^{1}H NMR (400 MHz, THF-d_8, 298 K): δ 6.92 (s, 1H), 6.48 (d, J = 9.0, 1H), 6.44 (d, J = 8.9, 1H), 6.35 (s, 1H), 6.04 (d, J = 9.0 Hz, 1H), 6.00 (d, J = 8.9 Hz, 1H), 3.64 – 3.59 (m, 4H), 3.49 (s, 36H), 3.45 (d, J = 2.9 Hz, 1H), 3.30 (d, J = 1.6 Hz, 1H), 1.81 – 1.75 (m, 3H), 1.35 (s, 2H), 1.18 (d, J = 8.9 Hz, 18H), 1.15 (d, J = 9.5 Hz, 18H) ppm.

^{13}C{^{1}H} APT NMR (101 MHz, THF-d_8, 298 K) δ 168.24 (d, J = 10.7 Hz), 168.06 (d, J = 6.3 Hz), 153.70, 152.76, 140.37, 130.74, 130.18, 122.93, 121.37, 120.25, 117.26, 116.88, 110.46, 71.34, 69.44 (d, J = 36.4 Hz), 66.11 (d, J = 48.6 Hz), 33.61, 33.46, 30.62, 30.54, -9.72 (d, J = 32.2 Hz) ppm.

^{31}P{^{1}H} NMR (162 MHz, THF-d_8, 298 K): δ 15.78 (bs), 2.52 (bs) ppm.

IR-ATR (cm^{-1}): 2945 (m), 2866 (m), 2196 (w), 1615 (m), 1580 (m), 1496 (w), 1464 (s), 1351 (m), 1250 (m), 1218 (w), 1109 (s), 961 (m), 813 (m), 635 (w).

Anal. Calcd. For $C_{50}H_{82}Cu_2KN_3O_{7.5}P_2$: C, 55.95; H, 7.70; N, 3.91 %. Found C, 49.99; H, 7.23; N, 3.56 %. The calculated values are provided for 1.5 coordinated THF molecules.

The reactive nature of this compound precluded obtaining a satisfactory result for the elemental analysis. The values that are found are consistent with the incorporation of 3 eq. of H_2O and 2 eq. of O_2, (calcd: C, 50.07; H 7.4; N, 3.50) reflecting the reactive nature of the compound.

*The purity of **3** was quantified by ^{1}H qNMR spectroscopy using trimethoxybenzene as internal standard as 79.8%. The other impurities are: KHMDS (5.0 %), HHMDS (4.2 %) and trace amounts of residual solvent (11.0%).

S2.10 *In situ* generation of ^{tBu}**PN-NP***

A solution of KHMDS (20.1 mg, 0.1 mmol, 1 eq.) and 18-crown-6 (26.6 mg, 0.1 mmol, 1 eq.) in THF (8 mL) was added dropwise to a stirred, brown solution of ^{tBu}**PN-NP** (50 mg, 0.1 mmol, 1 eq.) in THF (8 mL), during which the reaction mixture turned blue. The reaction mixture was stirred at room temperature for 1 h and an aliquot was taken for ^{1}H NMR analysis, confirming full conversion to ^{tBu}**PN-NP*.** The volatiles can be removed under reduced pressure to yield ^{tBu}**PN-NP*** as a blue powder, which slowly decomposes over the course of days.

^{1}H NMR (400 MHz, THF-h_8, 298 K): δ 7.43 (dd, *J* = 9.4, 4.9 Hz, 1H), 7.24 (d, *J* = 8.1 Hz, 1H), 6.67 (s, 1H), 6.57 (s, 1H), 6.54 (d, *J* = 8.0 Hz, 1H), 6.44 (d, *J* = 9.5 Hz, 1H), 4.34 (dd, *J* = 8.6, 1.2 Hz, 1H), 2.89 (d, *J* = 2.7 Hz, 2H), 1.08 (dd, *J* = 19.9, 10.4 Hz, 36H) ppm.

^{31}P{^{1}H} NMR (162 MHz, THF-h_8, 298 K): δ 31.75 (s), 13.98 (s) ppm.

S2.11 *In situ* generation of ^{tBu}**PN-NP****

A solution of KHMDS (40.2 mg, 0.2 mmol, 16 eq.) and 18-crown-6 (53.2 mg, 0.2 mmol, 16 eq.) in THF (8 mL) was added dropwise to a stirred, blue solution of ^{tBu}**PN-NP*** (10 mg, 0.012 mmol, 1 eq.) in THF (8 mL), during which the reaction mixture turned red. The reaction mixture was stirred at room temperature for 1 h and an aliquot was taken for ^{1}H NMR analysis, confirming full conversion to ^{tBu}**PN-NP**.**

Alternative method:

A solution of BnK (67% purity, 6.25 mg, 0.032 mmol, 2 eq.) and THF (5 mL) was added dropwise to a stirred, brown solution of ^{tBu}**PN-NP** (8.0 mg, 0.016 mmol, 1 eq.) in THF (8 mL), during which the reaction mixture turned red. The reaction mixture was stirred at room temperature for 1 h and an aliquot was taken for ^{1}H NMR analysis, confirming full conversion to ^{tBu}**PN-NP**.**

^{1}H NMR (400 MHz, THF-h_8, 298 K): δ 7.22 (dd, *J* = 9.2, 4.2 Hz, 2H), 6.53 (d, *J* = 9.2 Hz, 2H), 6.40 (s, 1H), 5.99 (s, 1H), 4.09 (dd, *J* = 8.9, 1.0 Hz, 2H), 1.31 (d, *J* = 10.2 Hz, 36H) ppm.

^{31}P{^{1}H} NMR (162 MHz, THF-h_8, 298 K): δ 14.93 (s) ppm.

S3. 1D NMR spectra

S3.1 ^{tBu}PN-NP

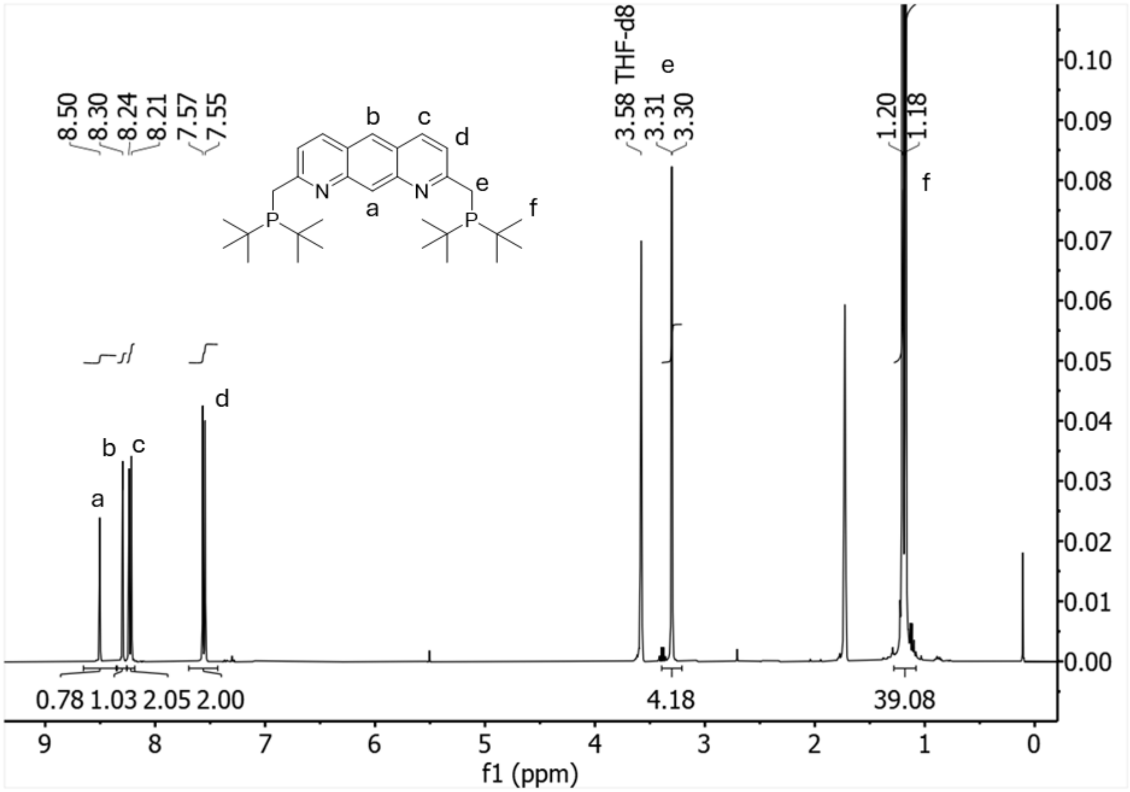

Figure S1: ^{1}H NMR spectrum of ^{tBu}PN-NP in THF-d_8 at 298 K.

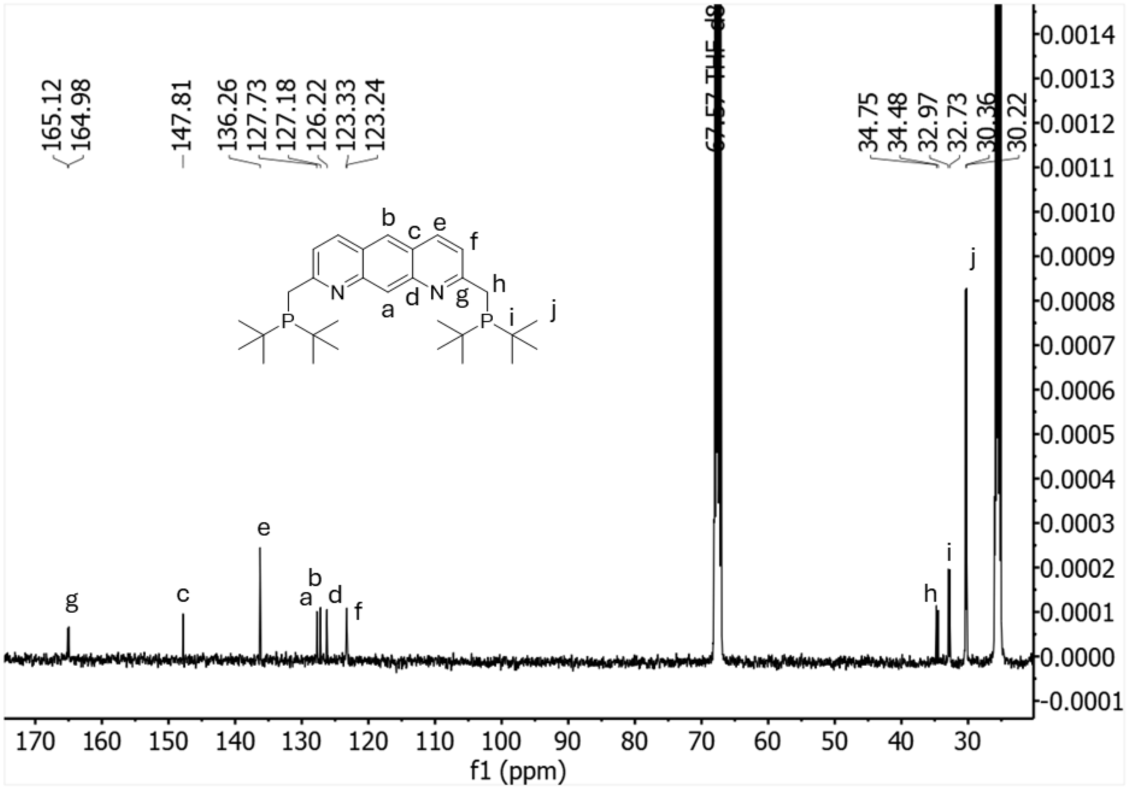

Figure S2: $^{13}C\{^{1}H\}$ NMR spectrum of ^{tBu}PN-NP in THF-d_8 at 298 K.

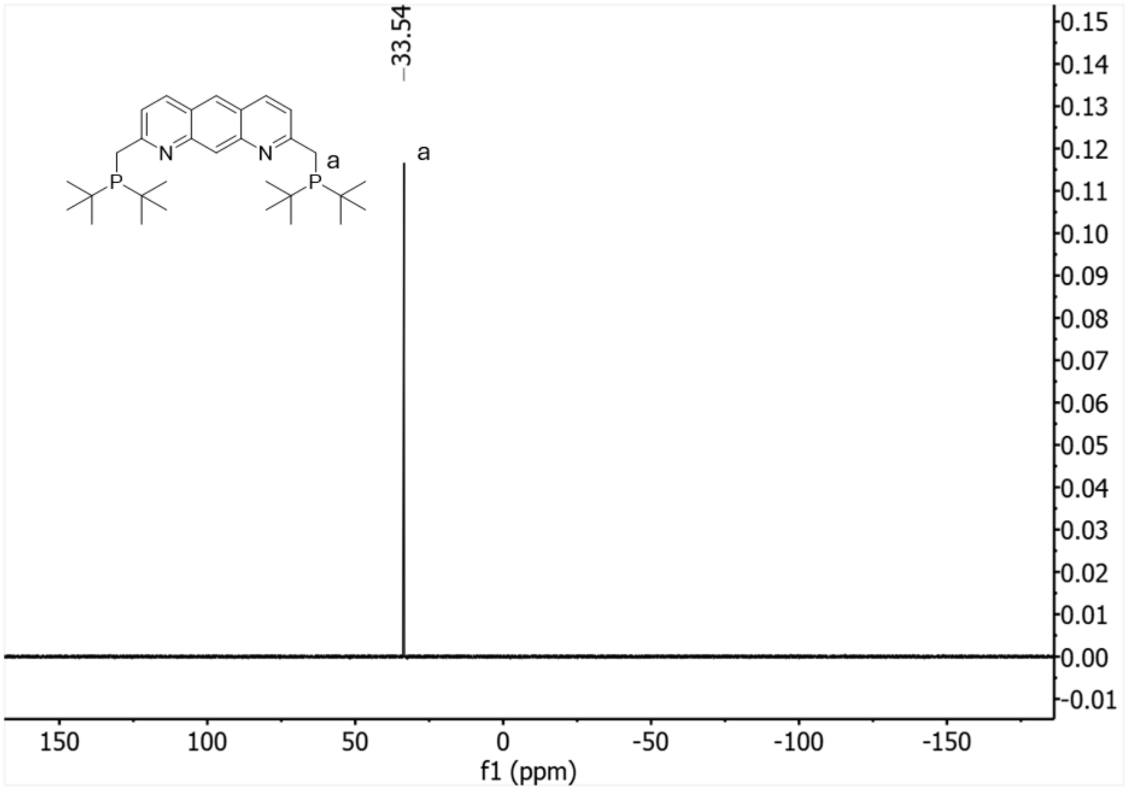

Figure S3: $^{31}P\{^{1}H\}$ NMR spectrum of ^{tBu}PN-NP in THF-d_8 at 298 K.

S3.2 [$Cu_2(MeCN)_2$ ^{tBu}PN-NP][$2PF_6$](**1**)

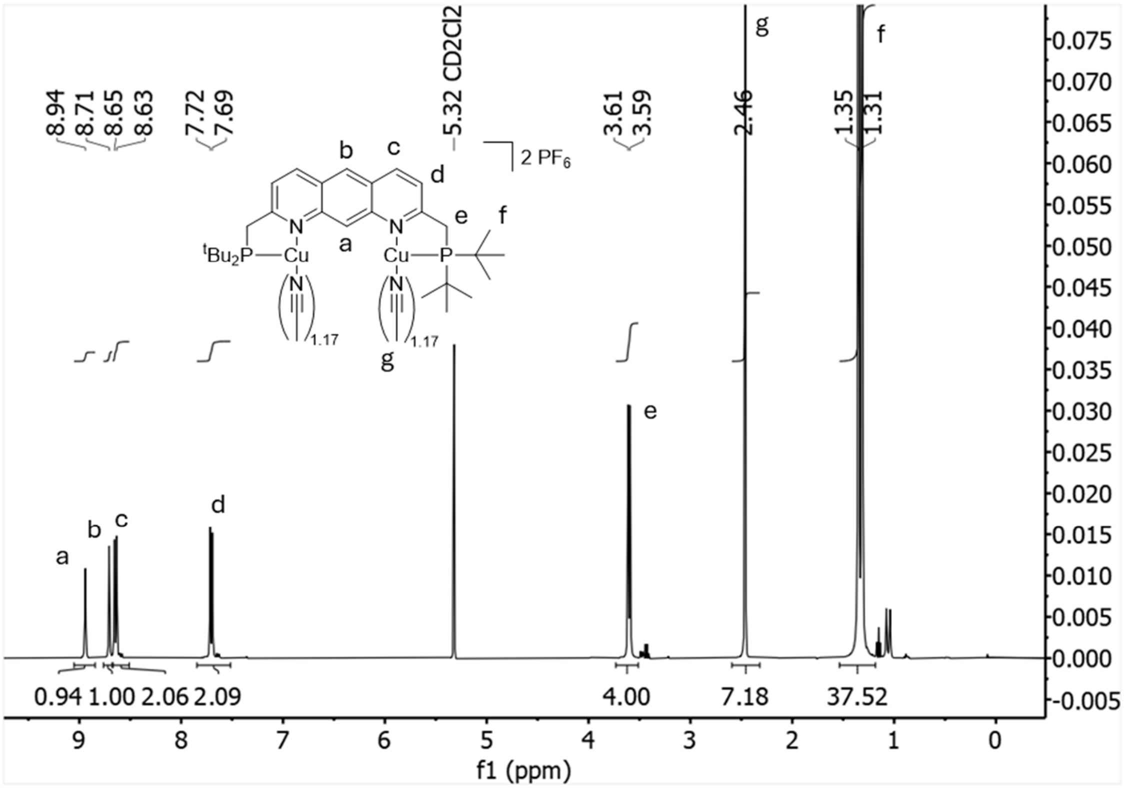

Figure S4: ^{1}H NMR spectrum of **1** in CD_2Cl_2 at 298 K.

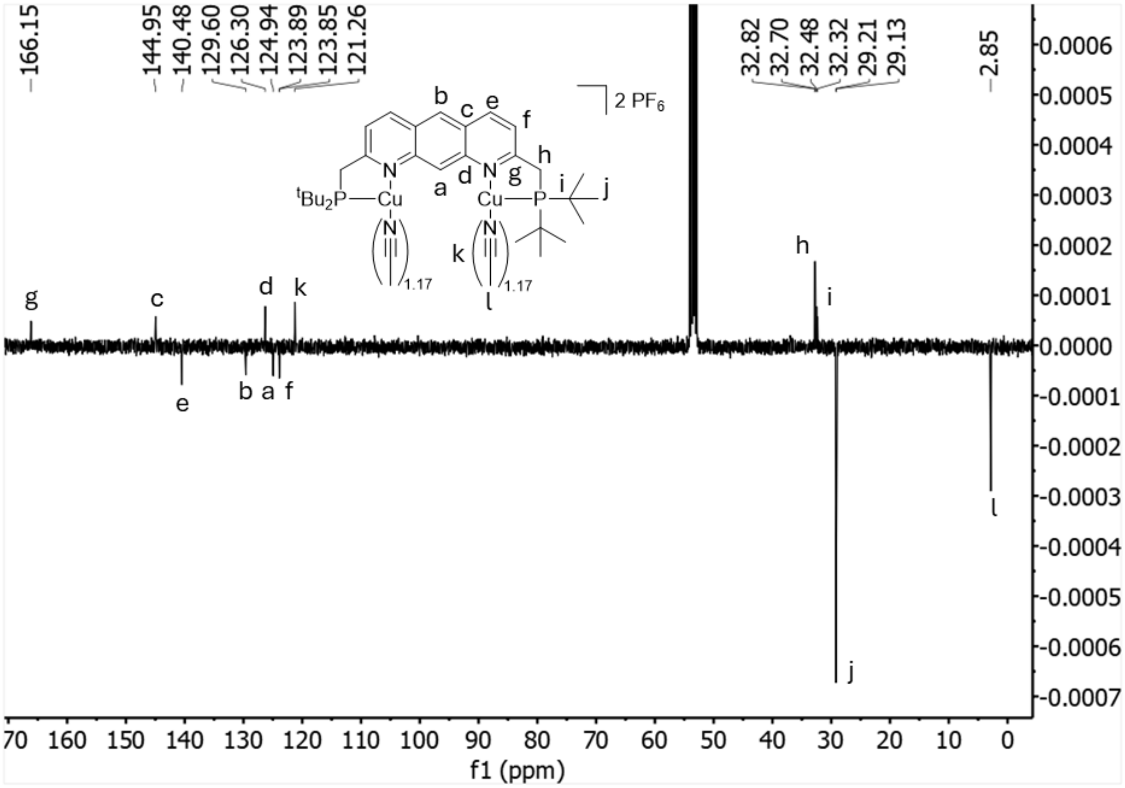

Figure S5: $^{13}C\{^{1}H\}$ APT NMR spectrum of **1** in CD_2Cl_2 at 298 K.

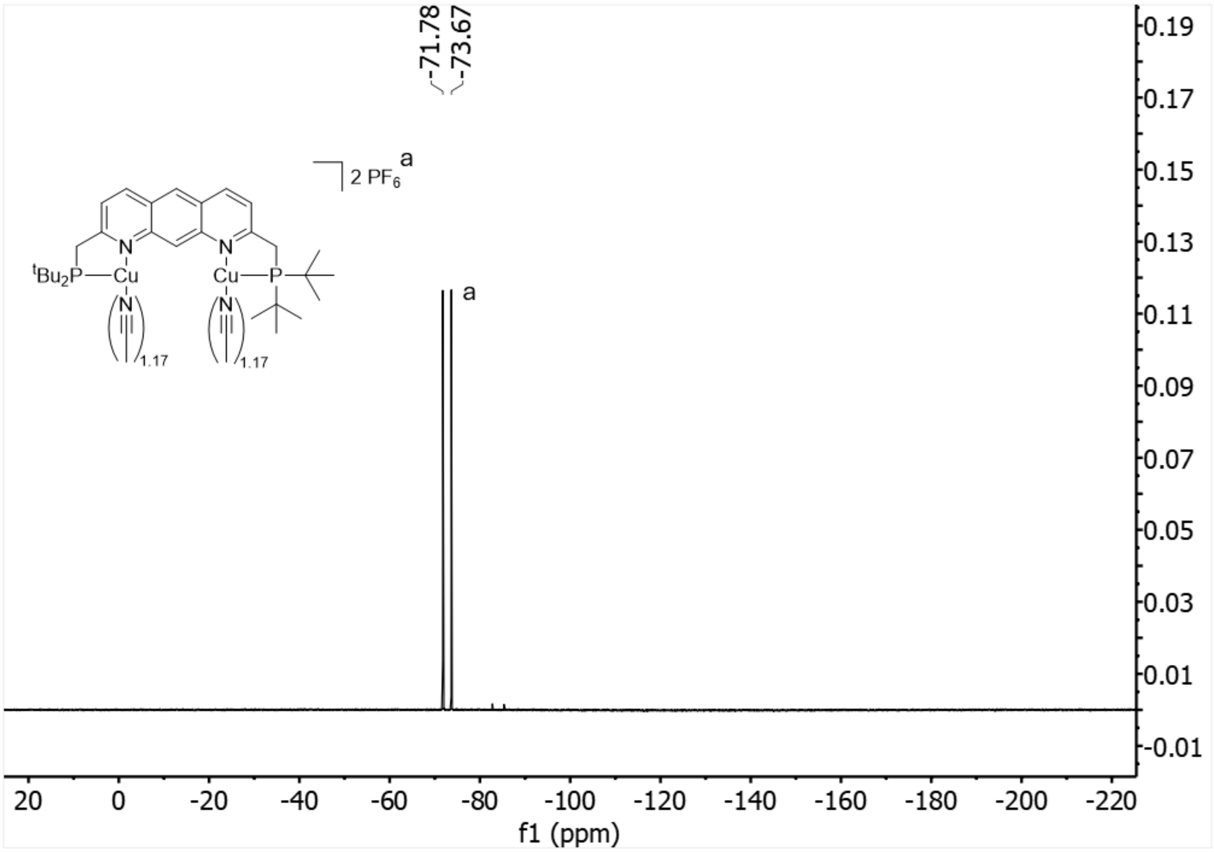

Figure S6: ^{19}F NMR spectrum of **1** in CD_2Cl_2 at 298 K.

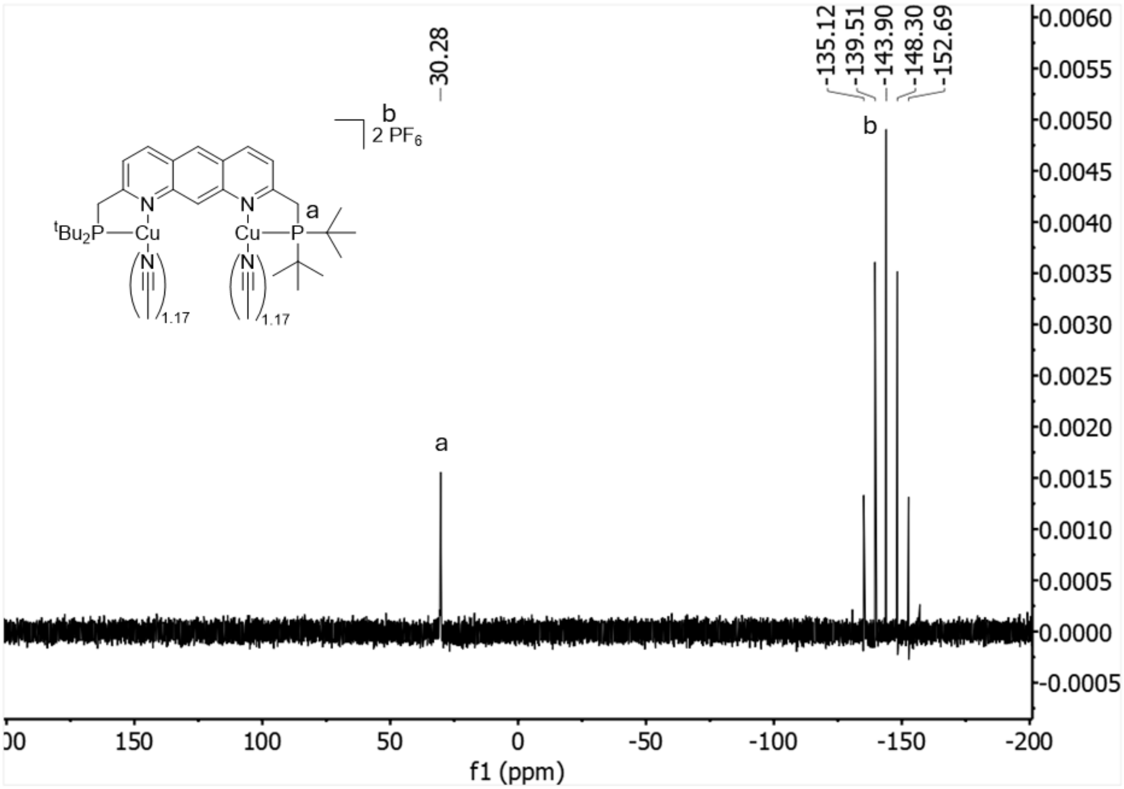

Figure S7: ^{31}P NMR spectrum of **1** in CD_2Cl_2 at 298 K.

S3.3 $[Cu_2(\mu\text{-}MeCN)^{tBu}PN\text{-}NP^*]$ ($\mathbf{2^{a*}}$ and $\mathbf{2^{b*}}$)

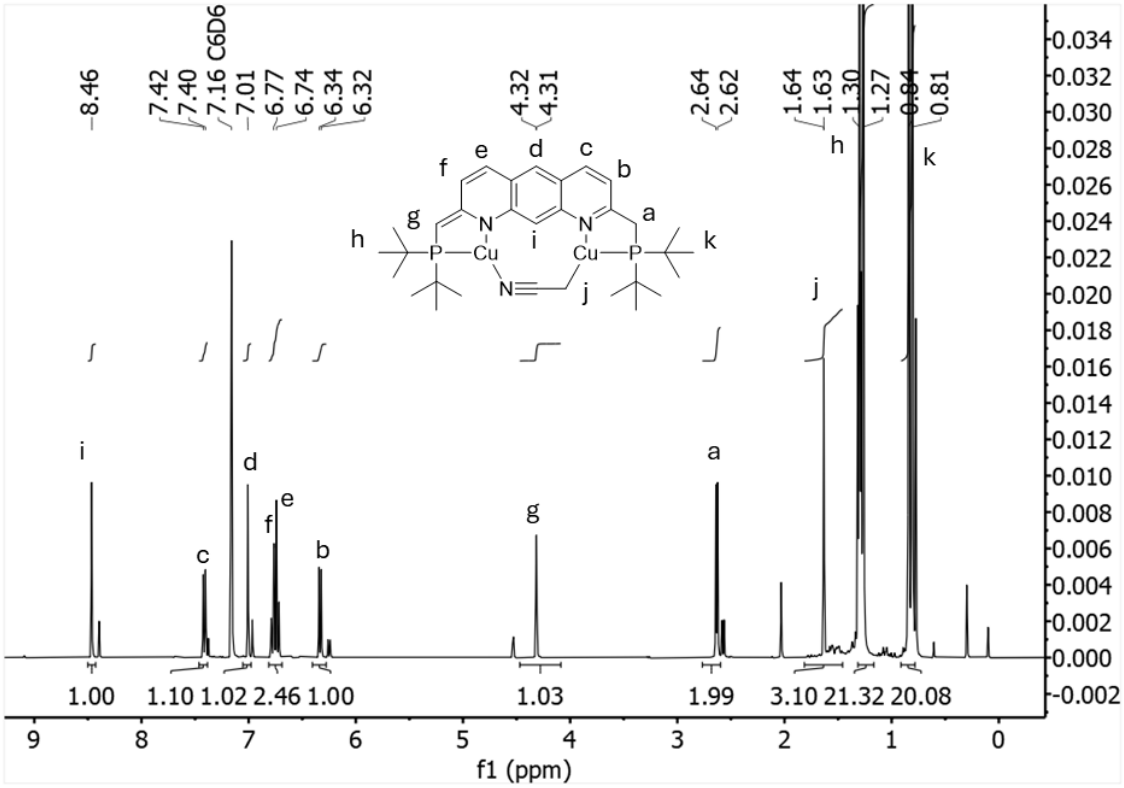

Figure S8: ^1H NMR spectrum of $\mathbf{2^{a*}}$ in C_6D_6 at 298 K.

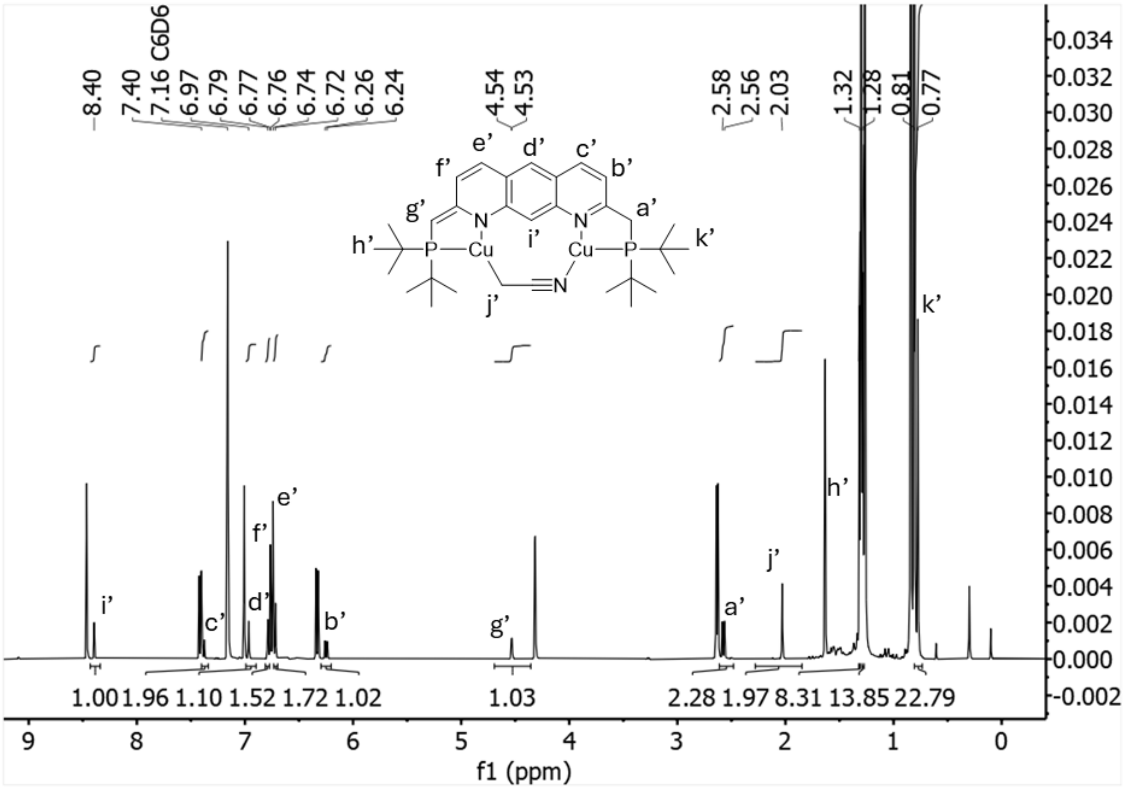

Figure S9: ^{1}H NMR spectrum of $\mathbf{2^{b*}}$ in C_6D_6 at 298 K.

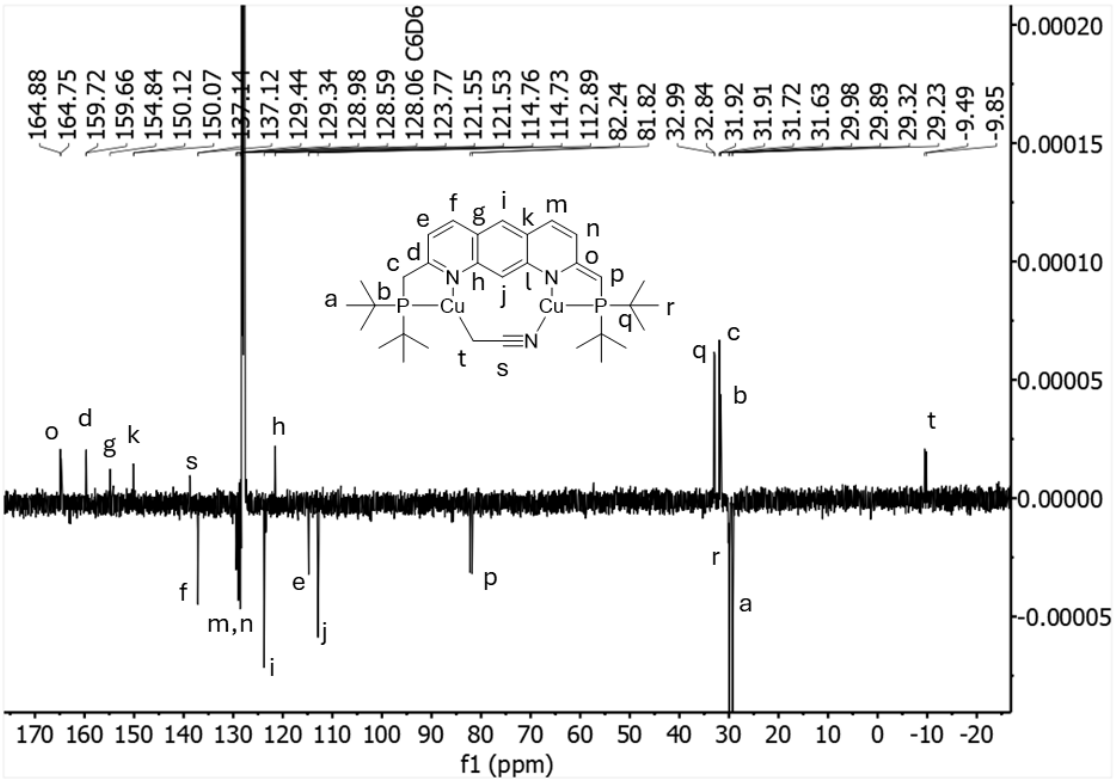

Figure S10: $^{13}C\{^{1}H\}$ NMR spectrum of $\mathbf{2^{a*}}$ in C_6D_6 at 298 K.

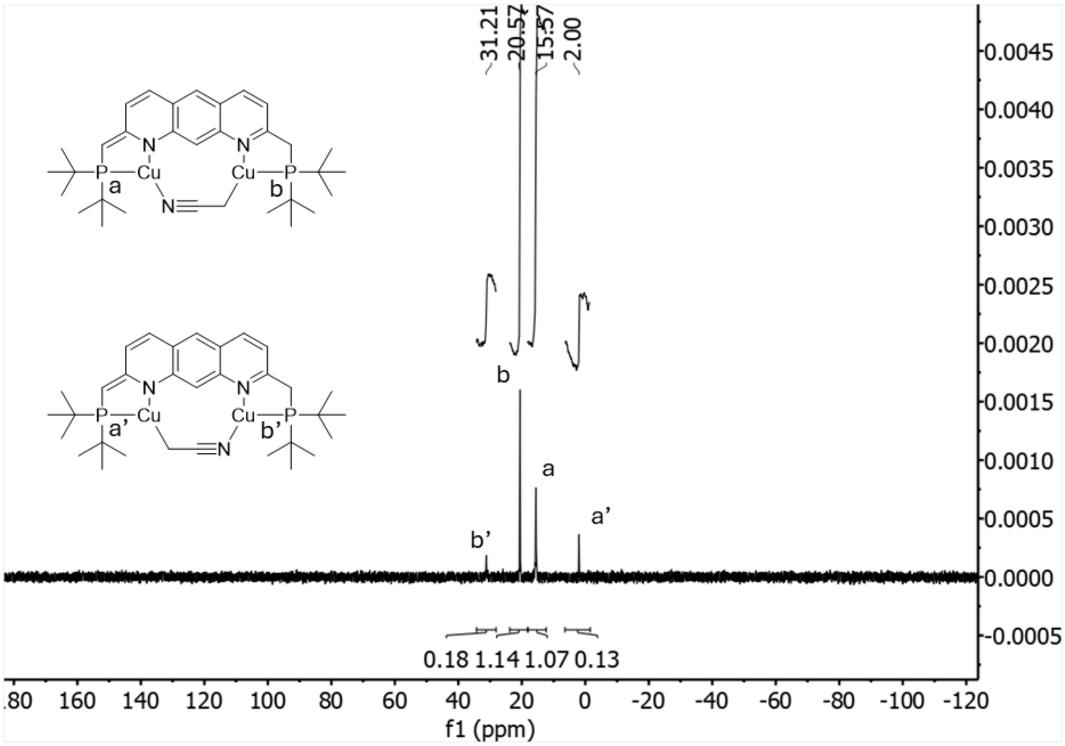

Figure S11: $^{31}P\{^{1}H\}$ NMR spectrum of $\mathbf{2^{a*}}$ and $\mathbf{2^{b*}}$ in C_6D_6 at 298 K.

S3.4 [Cu_2(μ-MeCN)^{tBu}PN-NP**][K(18-crown-6)] (**3**)

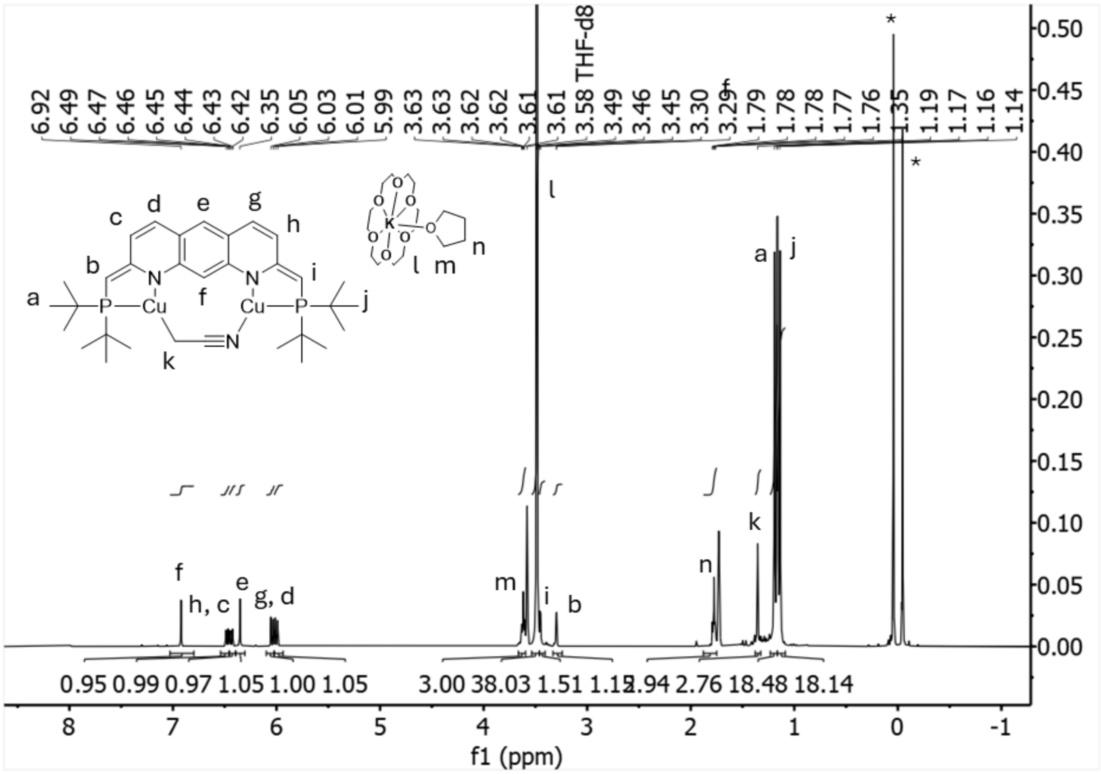

Figure S11: ^{1}H NMR spectrum of **3** in THF-d_8 at 298 K.

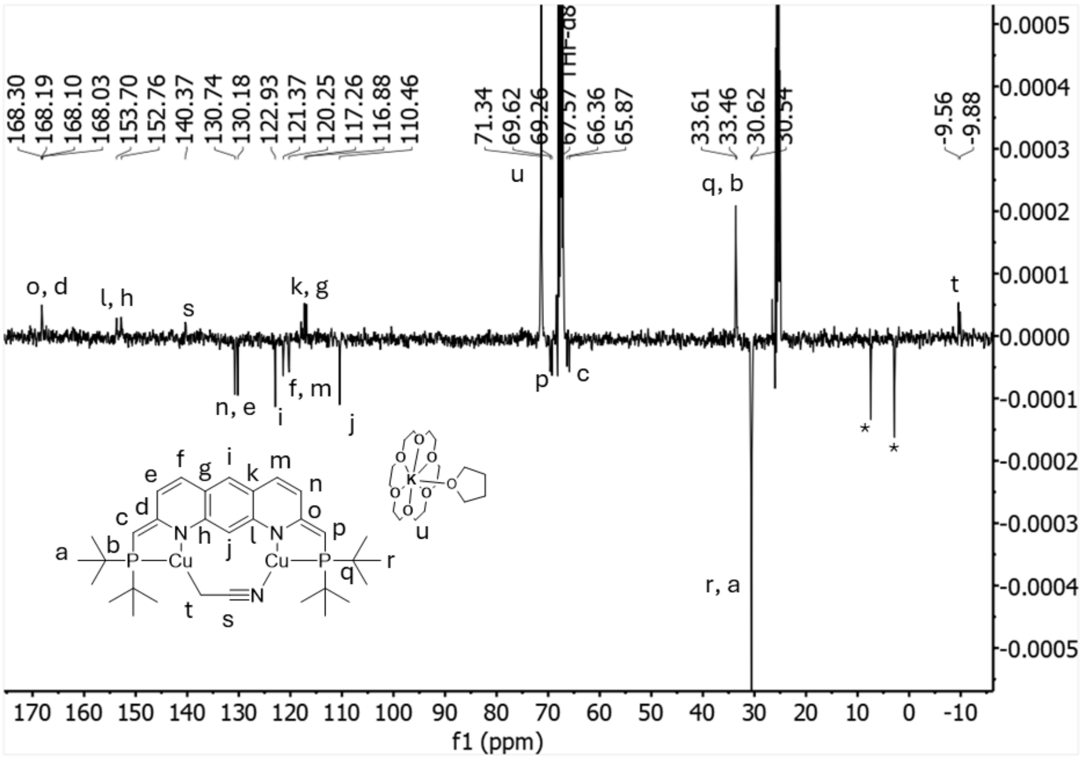

Figure S12: ^{13}C{^{1}H} APT NMR spectrum of **3** in THF-d_8 at 298 K.

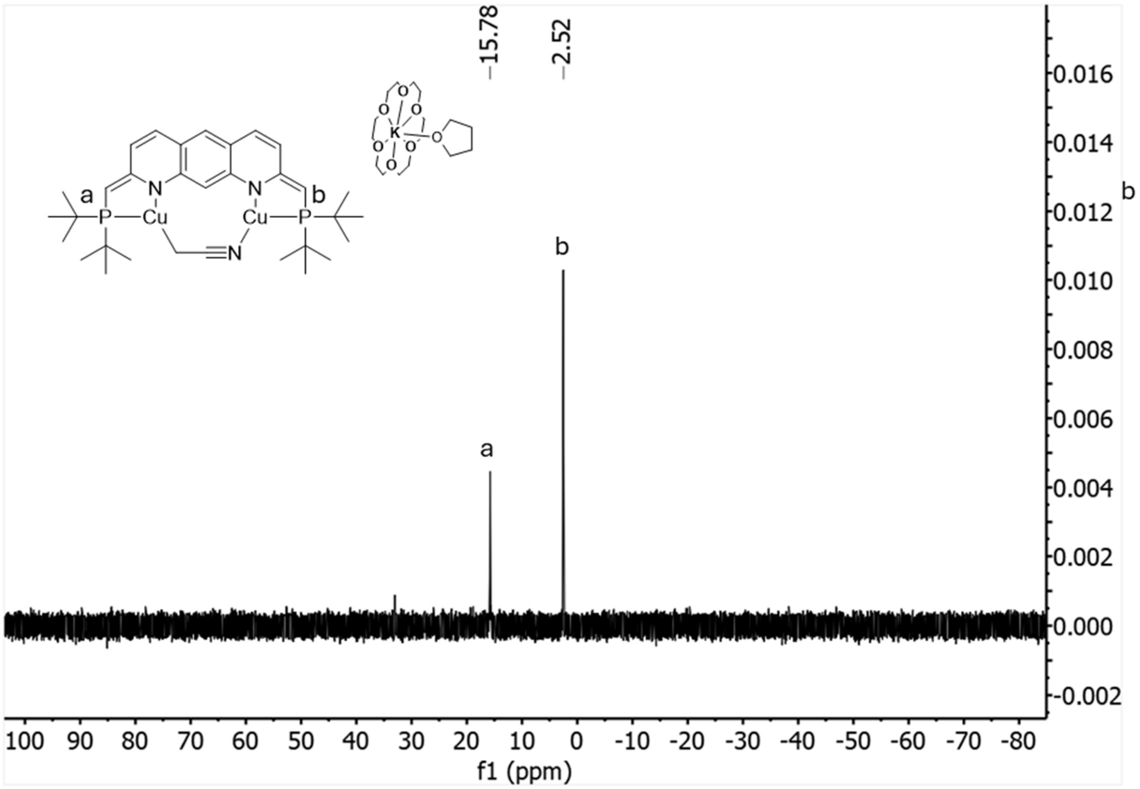

Figure S13: $^{31}P\{^{1}H\}$ NMR spectrum of **3** in THF-d_8 at 298 K.

S3.5 ^{tBu}PN-NP*

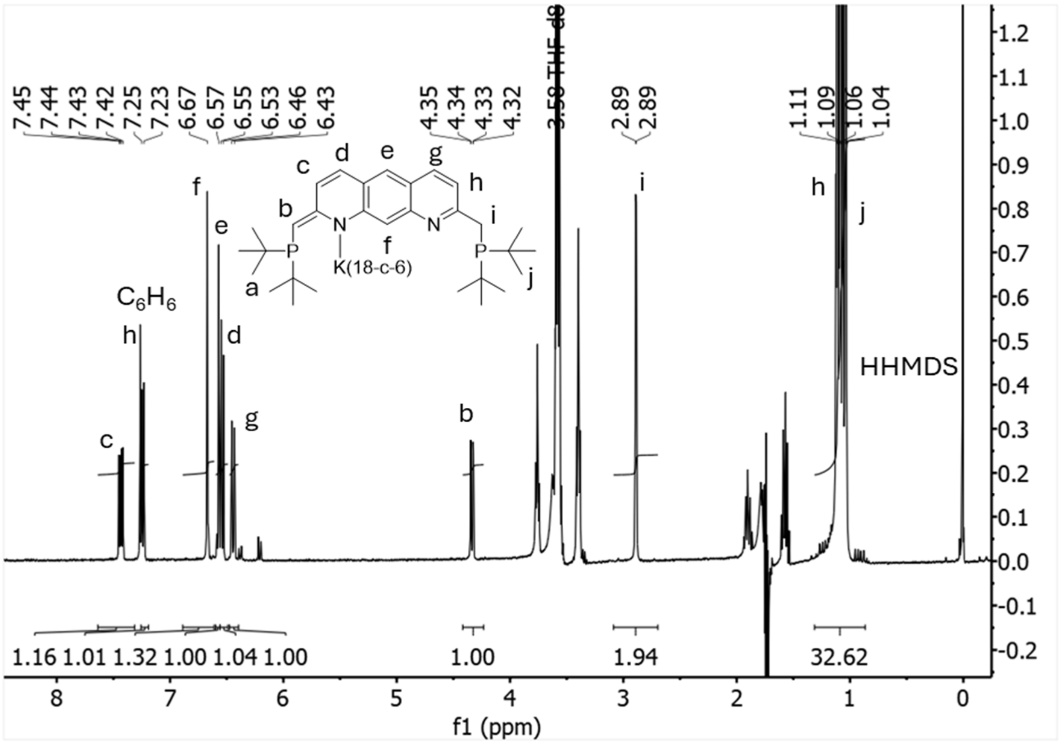

Figure S14: Solvent suppressed ^{1}H NMR spectrum of **^{tBu}PN-NP*** in THF-h_8 at 298 K.

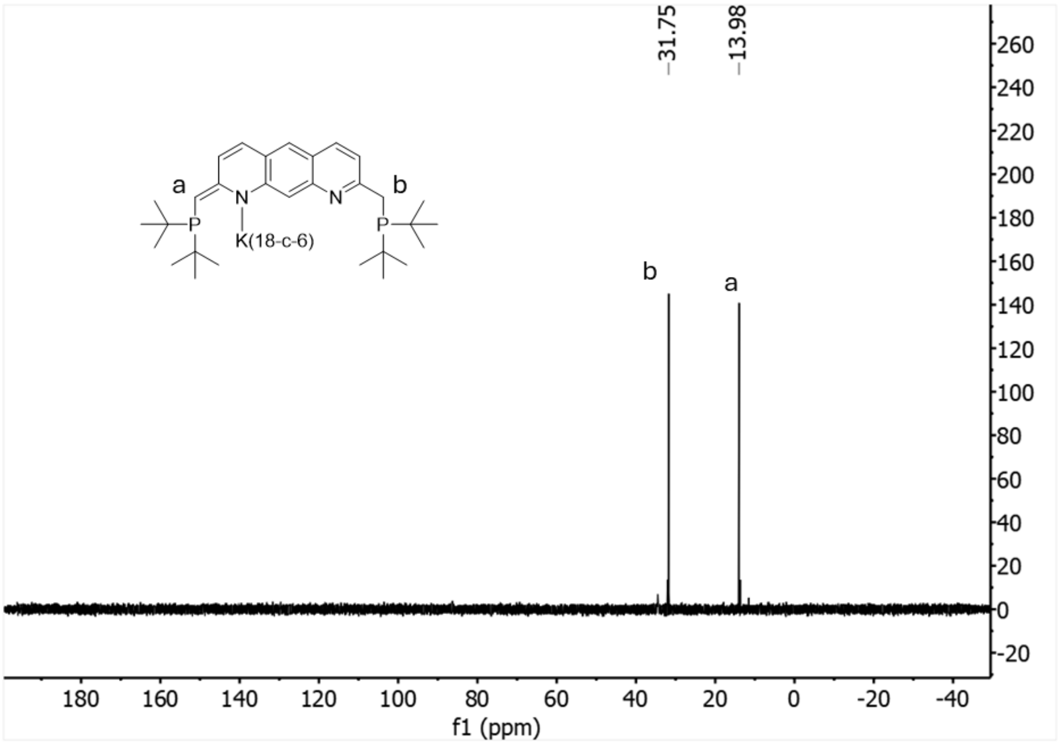

Figure S15: $^{31}P\{^1H\}$ NMR spectrum of **^{tBu}PN-NP*** in THF-h_8 at 298 K.

S3.6 ^{tBu}PN-NP**

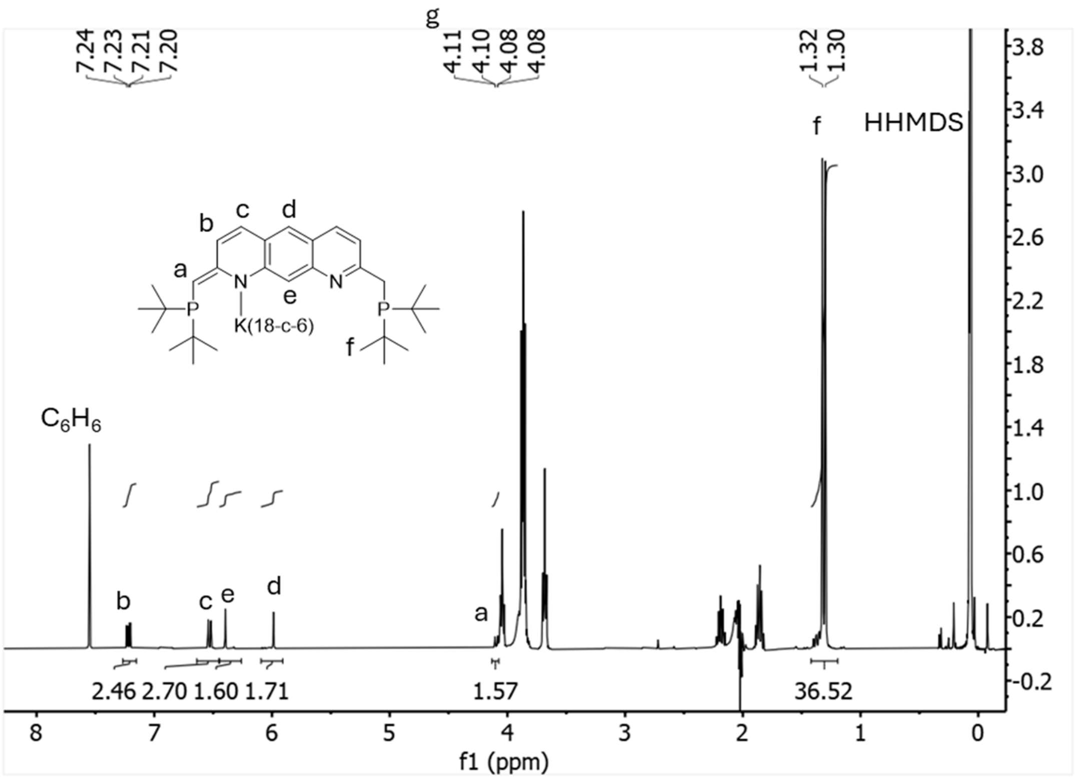

Figure S16: Solvent suppressed ^{1}H NMR spectrum of **^{tBu}PN-NP**** in THF-h_8 at 298 K.

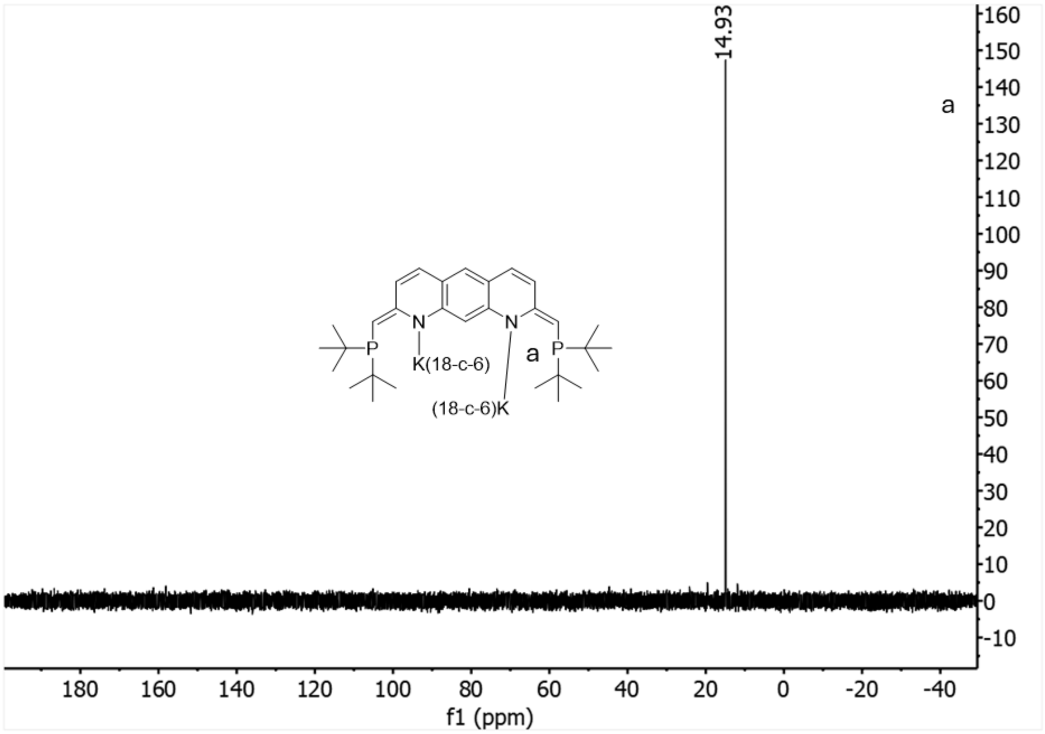

Figure S15: ^{31}P{^{1}H} NMR spectrum of **^{tBu}PN-NP**** in THF-h_8 at 298 K.

S4. IR spectra

S4.1 ^{tBu}PN-NP

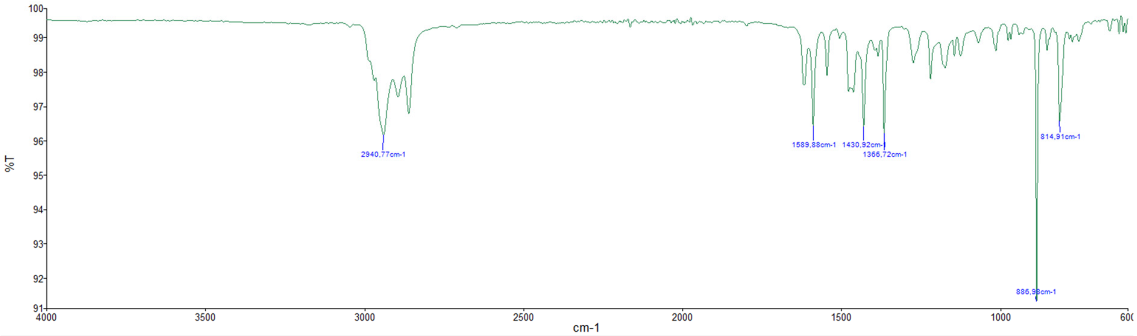

Figure S16: ATR-IR spectrum of ^{tBu}**PN-NP** at 298 K.

S4.2 [Cu_2(MeCN)$_2$^{tBu}PN-NP][$2PF_6$](**1**)

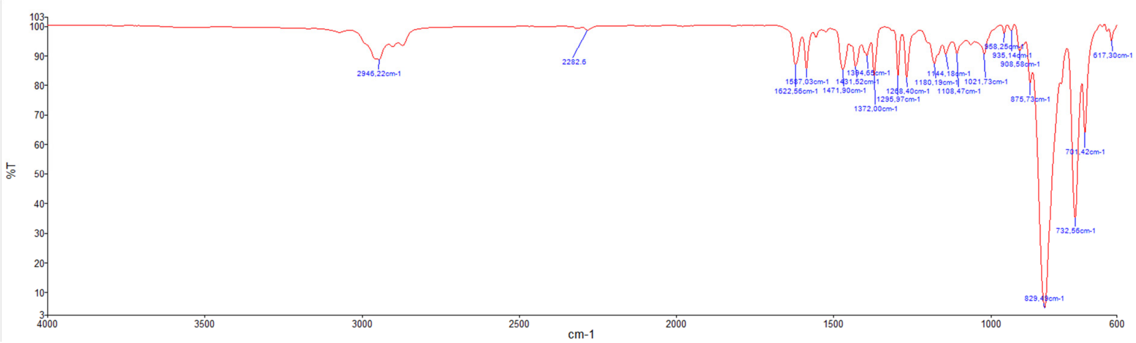

Figure S17: ATR-IR spectrum of [Cu_2(MeCN)$_2$^{tBu}PN-NP][$2PF_6$](**1**) at 298 K.

S4.3 [Cu_2(µ-MeCN)^{tBu}PN-NP*] (**2**^{a}***** and **2**^{b}*****)

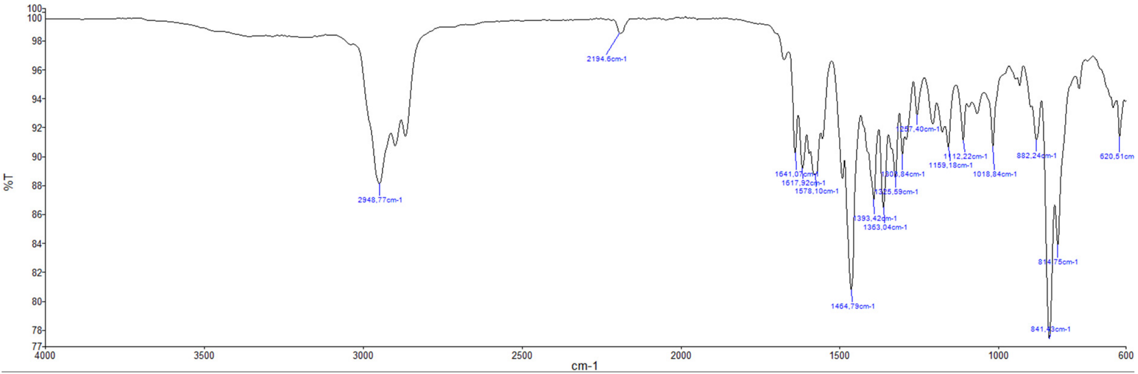

Figure S18: ATR-IR spectrum of [Cu_2(µ-MeCN)^{tBu}PN-NP*] (**2**^{a}***** and **2**^{b}*****) at 298 K.

S4.4 [Cu_2(μ-MeCN)^{tBu}PN-NP**][K(18-crown-6)] (**3**)

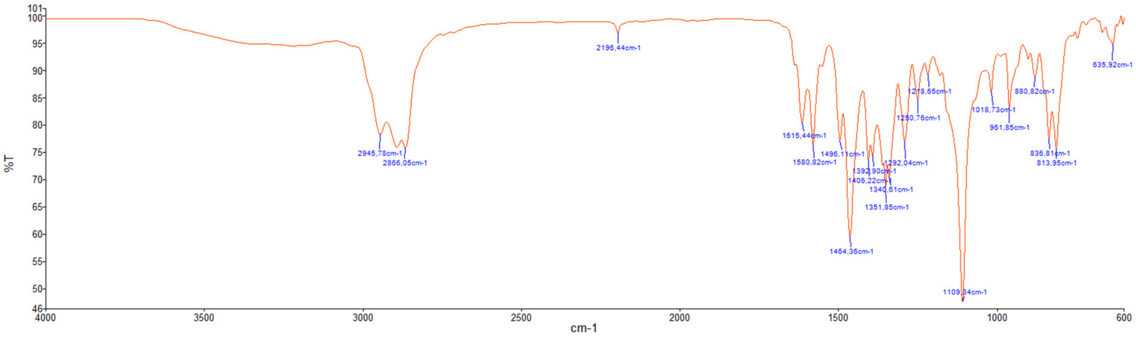

Figure S19: ATR-IR spectrum of [Cu_2(μ-MeCN)^{tBu}PN-NP**][K(18-crown-6)] (**3**) at 298 K.

S5 HR-MS spectrum of ^{tBu}PN-NP.

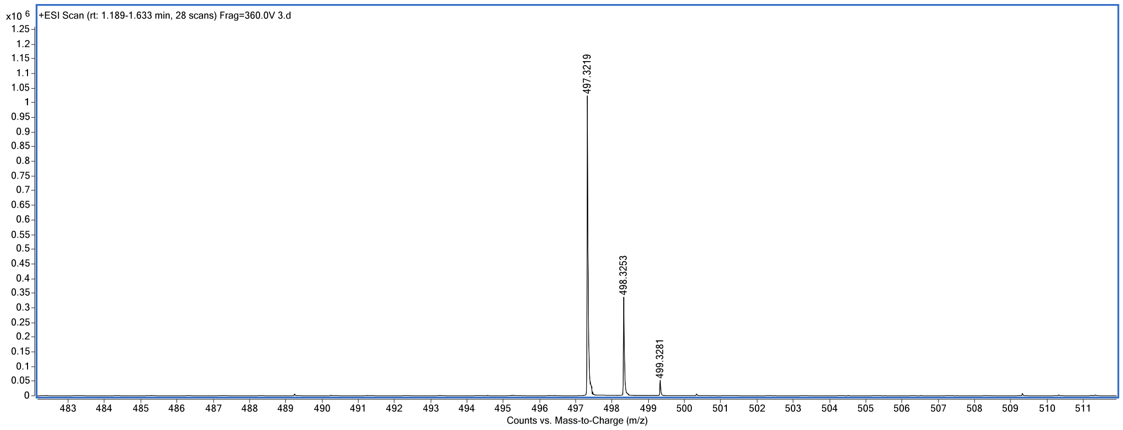

Figure S20: HR-MS spectrum of ^{tBu}**PN-NP**.

S6. X-ray crystal structures:

S6.1 X-ray crystal structure determination of (**1**)

$[C_{36}H_{55}Cu_2N_5P_2](PF_6)_2$ + disordered solvent, Fw = 1036.81*, orange block, 0.52 × 0.45 × 0.44 mm^3, monoclinic, I2/a (no. 15), a = 30.8192(5), b = 9.9199(2), c = 35.6101(6) Å, β = 111.319(1) °, V = 10141.8(3) Å^3, Z = 8, D_x = 1.358 g/cm^3*, μ = 1.04 mm^{-1}*. The diffraction experiment was performed on a Bruker Kappa ApexII diffractometer with sealed tube and Triumph monochromator (λ = 0.71073 Å) at a temperature of 150(2) K up to a resolution of $(\sin \theta/\lambda)_{max}$ = 0.65 Å^{-1}. Intensity integration was performed with the Eval15 software[4]. The presence of significant diffuse scattering was ignored during the intensity integration. A multi-scan absorption correction and scaling was performed with SADABS[5] (correction range 0.69-0.75). A total of 89981 reflections was measured, 11647 reflections were unique (R_{int} = 0.028), 8983 reflections were observed [I>2σ(I)]. The structure was solved with dual-space methods using SHELXT.[6] The structure contains large voids (1076 Å^3/unit cell) filled with severely disordered *n*-pentane molecules. Their contribution to the structure factors was secured by back-Fourier transformation using SQUEEZE[7], resulting in 364 electrons/unit cell. Structure refinement was performed with SHELXL-2019[8] on F^2 of all reflections. Non-hydrogen atoms were refined freely with anisotropic displacement parameters. The coordinated acetonitrile molecules were disordered between the two copper centers, and one PF_6 anion was orientationally disordered. Hydrogen atoms were introduced in calculated positions and refined with a riding model. 689 Parameters were refined with 1065 restraints (geometry and displacement parameters of the disordered moieties). R1/wR2 [I > 2σ(I)]: 0.0565 / 0.1691, R1/wR2 [all refl.]: 0.0700 / 0.1801. S = 1.087. Residual electron density between -1.05 and 2.02 e/Å^3. Geometry calculations and checking for higher symmetry was performed with the PLATON program.[9]

* Derived values do not contain the contribution of the disordered solvent.

S6.2 X-ray crystal structure determination of (**3**)

$[C_{20}H_{40}KO_8][C_{32}H_{46}Cu_2N_3P_2] \cdot C_4H_8O$, Fw = 1181.46, red block, 0.62 × 0.25 × 0.14 mm^3, monoclinic, I2/a (no. 15), a = 18.1167(7), b = 15.6991(8), c = 43.924(2) Å, β = 98.178(2)°, V = 12365.7(10) Å^3, Z = 8, D_x = 1.269 g/cm^3, μ = 0.86 mm^{-1}. The diffraction experiment was performed on a Bruker Kappa ApexII diffractometer with sealed tube and Triumph monochromator (λ = 0.71073 Å) at a temperature of 150(2) K up to a resolution of $(\sin \theta/\lambda)_{max}$ = 0.61 Å^{-1}. The crystal appeared to be very fragmented. Four orientation matrices were used for the intensity integration with the Eval15 software[4]. Only non-overlapping reflections were used for the structure solution and refinement. A multi-scan absorption correction and scaling was performed with SADABS[5] (correction range 0.55-0.75). A total of 76932 reflections was measured, 11531 reflections were unique (R_{int} = 0.080), 7596 reflections were observed [I>2σ(I)]. The structure was solved with dual-space methods in SHELXT[6]. Structure refinement was performed with SHELXL-2019[8] on F^2 of all reflections. Non-hydrogen atoms were refined freely with anisotropic displacement parameters. One of the independent cations and the non-coordinated THF molecule were refined with disorder models. Hydrogen atoms of the copper complex were located in difference Fourier maps. All other hydrogen atoms were introduced in calculated positions. All hydrogen atoms were refined with a riding model. 845 Parameters were refined with 1074 restraints (geometry and displacement parameters of the disordered moieties). R1/wR2 [I > 2σ(I)]: 0.0506 / 0.1235. R1/wR2 [all refl.]: 0.0880 / 0.1463. S = 1.013. Residual electron density between -0.35 and 0.67 e/Å^3. Geometry calculations and checking for higher symmetry was performed with the PLATON program.[9]

S6.3 Metric parameters of (**1**) and (**3**)

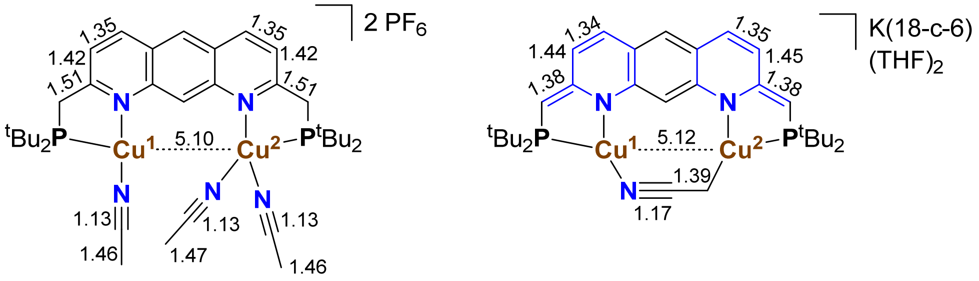

1 **3**

Figure S21: Schematic representation of the metric parameters in complexes **1** and **3** for facile comparison of (a) the change in the ligand carbon-carbon bond lengths upon full dearomatization (**3**) and (b) the change in the cyanomethyl carbon-carbon lengths upon acetonitrile activation.

S7. References

(1) Quast, H.; Schön, N. Synthese und Reaktionen einiger Pyrido [3, 2-g] chinoline (1, 8-Diazaanthracene). *Liebigs Annalen der Chemie* **1984**, *1984* (1), 133-146.

(2) Berlin, A.; Bradamante, S.; Ferraccioli, R.; Pagani, G.; Sannicolo, F. Expeditious synthesis of dihydrobenzo-[2, 1-b: 3, 4-b′]-,[1, 2-b: 5, 4-b′], and [1, 2-b: 4, 5-b′]-dipyrroles. *Journal of the Chemical Society, Chemical Communications* **1987**, (15), 1176-1177.

(3) Wang, R.; Liu, H.; Li, J.; Tian, J.; Li, Z.; Zhao, Y. Solid-State Photodimerization of Azaanthracene Derivative Based on a [4+ 4] Cycloaddition. *Asian Journal of Organic Chemistry* **2018**, *7* (5), 906-909.

(4) Schreurs, A. M. M.; Xian, X.; Kroon-Batenburg, L. M. J. EVAL15: a diffraction data integration method based on ab initio predicted profiles. *Journal of Applied Crystallography* **2010**, *43*, 70–82.

(5) Krause, L.; Herbst-Irmer, R.; Sheldrick, G. M.; Stalke, D. Comparison of silver and molybdenum microfocus X-ray sources for single-crystal structure determination. *Journal of Applied Crystallography* **2015**, *48*, 3–10.

(6) Sheldrick, G. M. SHELXT – Integrated space-group and crystal-structure determination. *Acta Crystallographica Section A: Foundations and Advances* **2015**, *A71*, 3–8.

(7) Spek, A. L. PLATON SQUEEZE: a tool for the calculation of the disordered solvent contribution to the calculated structure factors. *Acta Crystallographica Section C: Structural Chemistry* **2015**, *C71*, 9–18.

(8) Sheldrick, G. M. Crystal structure refinement with SHELXL. *Acta Crystallographica Section C: Structural Chemistry* **2015,** *C71*, 3–8.

(9) Spek, A. L. Structure validation in chemical crystallography. *Acta Crystallographica Section D: Biological Crystallography* 2009, *D65*, 148–155.
